# PTRF/Cavin-1 enhances chemo-resistance and promotes temozolomide efflux through extracellular vesicles in glioblastoma

**DOI:** 10.7150/thno.71763

**Published:** 2022-05-16

**Authors:** Eryan Yang, Lin Wang, Weili Jin, Xing Liu, Qixue Wang, Ye Wu, Yanli Tan, Yunfei Wang, Xiaoteng Cui, Jixing Zhao, Fei Tong, Biao Hong, Menglin Xiao, Xiaomin Liu, Chuan Fang, Chunsheng Kang

**Affiliations:** 1 Department of Neurosurgery, Tianjin Medical University General Hospital, Tianjin 300052, China; 2 Tianjin Neurological Institute, Key Laboratory of Post-neurotrauma Neuro-repair and Regeneration in Central Nervous System, Ministry of Education, Tianjin 300052, China; 3 Beijing Neurosurgical Institute, Beijing Tiantan Hospital, Capital Medical University, Beijing, 100050, China; 4 Department of Pathology, Hebei University School of Basic Medical Sciences, Baoding 071000, China; 5 Department of Pathology, Affiliated Hospital of Hebei University, Baoding 071000, China; 6 Department of Neurosurgery, Affiliated Hospital of Hebei University, Baoding 071000, China; 7 Key Laboratory of Precise Diagnosis and Treatment of Glioma in Hebei Province, Baoding 071000, China; 8 Department of Oncology, Gamma Knife Center, Department of Neurological Surgery, Tianjin Huanhu Hospital, Nankai University, Tianjin 300350, China; These authors contributed equally to this work: Eryan Yang, Lin Wang and Weili Jin

**Keywords:** Glioblastoma, temozolomide, PTRF, extracellular vesicles, chloroquine

## Abstract

**Background:** The concentration and duration of intracellular drugs have always been the key factors for determining the efficacy of the treatment. Efflux of chemotherapeutic drugs or anticancer agents is a major reason for multidrug resistance generation in cancer cells. The high expression of polymerase I and transcript release factor (PTRF) is correlated with a worse prognosis in glioma patients. However, the importance of PTRF on temozolomide (TMZ) resistance in glioblastoma (GBM) is poorly understood.

**Methods:** TCGA data analysis, CGGA data analysis, transmission electron microscopy (TEM), scanning electron microscopy (SEM), clone formation, cell counting kit-8 (cck-8), western blot (WB), immunofluorescence (IF), immunohistochemistry (IHC) and flow cytometry assays were performed to investigate the underlying mechanism and effect of PTRF on TMZ-resistance in a variety of GBM cell lines and GBM patient-derived xenograft (PDX) models. Clone formation, WB, IF, IHC and flow cytometry assays were performed to examine the efficacy of sequential therapy of TMZ followed by CQ in GBM cells and PDX models.

**Results:** The prognosis of GBM patients treated with TMZ was negatively correlated with PTRF expression. Our results reveal that PTRF knockdown significantly decrease proliferation and increase apoptosis in GBM after TMZ treatment. Moreover, PTRF contribute to TMZ-resistance by increasing TMZ efflux through extracellular vesicles (EVs). Furthermore, our results demonstrate that sequential therapy of TMZ followed by CQ significantly promotes the TMZ efficacy against GBM by increasing intracellular TMZ concentration ([TMZ]i).

**Conclusion:** This study highlights that PTRF can act as an independent biomarker to predict the prognosis of GBM patients after TMZ treatment and describes a new mechanism contributing to TMZ-resistance. In addition, this study may provide a novel idea for GBM therapy.

## Introduction

Glioblastoma multiforme (GBM) is the most commonly occurring malignant primary intracranial tumor with a high mortality rate in adults 1. Following GBM diagnosis, presently available conventional therapies include safe surgical removal of GBM tumor followed by the combination of radio- and chemotherapy. Temozolomide (TMZ) is an oral alkylating agent routinely prescribed to treat GBM. Although TMZ exhibits significant therapeutic potential against GBM during the early phase of the treatment, more than 40% of GBM patients gradually develop TMZ resistance, leading to worsening of the diseased condition and death 2, 3. Currently, overcoming TMZ-resistance remains a major challenge for GBM treatment. Therefore, searching for a reliable biomarker to predict TMZ efficacy and further exploring the underlying mechanisms responsible for TMZ-resistance in GBM patients are urgently needed.

Polymerase I and transcript release factor (PTRF), also known as cavin-1, together with caveolins, is closely associated with the formation and function of the caveolae 4. Our previous studies have revealed PTRF's direct involvement in the cell-derived exosome (EXO) formation and secretion to maintain the intercellular communication between glioma cells 5, 6. Moreover, EXOs act as transporters of regulatory DNA, RNA, and protein, thereby facilitating the signal exchange between the donor and recipient cells 7. Interestingly, breast cancer cell-derived EXOs have been shown to induce chemoresistance by reducing the intracellular drug concentration below the threshold level 8. Our previous study had revealed that high expression of PTRF triggered a cytoplasmic phospholipase A2 (cPLA2)-mediated phospholipid remodeling pathway that promoted GBM cell proliferation and suppressed tumor immune responses 9. PTRF might act as a positive modulator in multidrug resistant (MDR) of GBM patients and could modulate the sensitivity of GBM cells to some anticancer drugs 10. Since PTRF seems to be involved in promotion of glioma tumorigenesis and it has been correlated with drug resistance in glioma and breast cancer, we hypothesized that it could play a key role in TMZ resistance.

Chloroquine (CQ), a traditional antimalarial drug, is an oral drug with the capacity of nervous system penetration. CQ has been reported to sensitize cancer cells to chemotherapy by inhibiting autophagy 11, 12. GBM therapy; nonetheless due to the small sample size the reported advantage of CQ could be circumstantial 13. On the contrary, other studies have shown that autophagy inhibition is not enough to prevent tumor growth 14, 15. Interestingly, CQ administration changes the pH in lysosomes, resulting in the blockade of endocytosis and EXO release 16. Therefore, CQ might sensitize cancer cells to chemotherapy through yet unknown mechanisms. As discussed before, despite PTRF's close association with the endocytosis and exosome release processes, very little is known about the cross-talk between CQ and PTRF expression. Several reports have shown that CQ can effectively reverse the multidrug resistance of cancer cells by decreasing the outward transport of anticancer drugs 17, 18. These findings have aroused our interest to further explore the roles of CQ on the efficacy of TMZ in GBM.

In this study it is shown that PTRF can act as an independent predictive biomarker to evaluate the prognosis of TMZ treatment in GBM. PTRF knockout enhanced TMZ efficacy in GBM treatment and increased the intracellular TMZ concentration by decreasing extracellular vesicles-mediated TMZ efflux. In order to better facilitate clinical translation, a sequence of *in vitro* and *in vivo* experiments was performed to demonstrate that sequential therapy of TMZ followed by CQ inhibited glioma growth by increasing the intracellular TMZ concentration. Overall, our results suggest that sequential therapy of TMZ followed by CQ is a promising intervention strategy for GBM.

## Methods

### Data download and analysis

All the data were downloaded from publicly available TCGA (https://cancergenome.nih.gov) and CGGA (http://www.cgga.org.cn/) databases. X-tile plots are created to define the high PTRF and low PTRF groups 19. Differences in survival time between groups were visible by Kaplan-Meier survival analyses with a log-rank significance test. The univariate and multivariate Cox regression analyses were applied to evaluate the prognostic elements.

### Cell culture

TBD0220, a glioma cell line, was derived from a GBM patient who underwent surgery at Hebei University Affiliated Hospital 20. The TBD0220 were cultured in DMEM/F12 containing 10% FBS. The U87, T98G cell lines were purchased from ATCC (American Type Culture Collection, Manassas, VA, USA) and cultured in Dulbecco's modified Eagle medium (DMEM) containing 10% fetal bovine serum (FBS).

### EV isolation and characterization

We collected 10% FBS pre-depleted cell supernatant containing EVs. Next, 240 ml of supernatant were centrifugated to isolate EVs as previously reported 21. In brief, the cell supernatant was centrifugated at 500 g (10 min, 4 °C), then at 2,000g (15 min, 4 °C) to discard dead cells and cellular debris, and finally at 10,000 g (30 min, 4 °C) to collect large EVs (the size > 200 nm) and at 110,000 g (70 min, 4 °C) to collect small EVs (the size < 200 nm). Small EVs were resuspended in cold PBS, centrifuged at 110,000 g for another 70 min to collect small EVs, which were finally resuspended in 400 µL of PBS or RIPA buffer containing 1% PMSF. To ensure the quality of the extracted EVs, the particle size distribution and concentration of isolated large EVs and small EVs were calculated with the NanoSight system (NS300; Malvern instruments; UK). The protein concentration of EVs was measured with a BCA Protein Assay Kit (Solarbio) according to the manufacturer's protocols. The morphology of large EVs and small EVs was observed using transmission electron microscope (TEM).

### PTRF knockout U87 cell line

PTRF knockout U87 cell line was generated by CRISPR/Cas9 (shanghai integrated biotech solutions Inc.). CRISPR was designed using a CRISPR design web tool (http://crispr.mit.edu). The sgRNA (single guide RNA) sequences targeted by PTRF are listed in supplement Table S1. The sgRNAs were cloned into the pGK1.1/CRISPR/Cas9 vector (shanghai integrated biotech solutions Inc.). U87 cells were transfected with the sgRNA vectors, expanded and screened for mutations at nuclease target sites by PCR amplification of genomic sequences, followed by DNA sequencing and immunoblotting. The CRISPR cell line was clonal.

### siRNA transfection and lentiviral transduction

PTRF-eGFP lentivirus and luciferase vectors were purchased from GENECHEM (Shanghai, China) while siRNAs targeting PTRF were synthesized by GenePharma (Shanghai, China). Glioma cells were incubated in a six-well plate with a density of 70%-80%. The siRNA was transfected into TBD0220 and T98G cell lines using Lipofectamine 3000 (Invitrogen). The transfection efficiency was verified by qRT-PCR and western blotting assay at 48 h post-transfection. The siRNA sequences are listed in supplement table S2. The siRNA sequences for PTRF were obtained from GeneChem (Shanghai, China). Lentiviruses containing the PTRF-eGFP fusion protein were transduced into U87, U87-PTRF^ko^, TBD0220, and T98G cell lines. The positive PTRF-transfected cells were selected by puromycin for two weeks at 2 μg/ml.

### Colony formation and cell viability assays

For the colony formation assay, 300 cells were seeded in 6-well plates and cultured for 2 weeks after indicated treatments. Cells were then fixed with 4% paraformaldehyde (PFA) for 30 min and subsequently stained with crystal violet for another 30 min followed by washing with water. The number of clones formed more than 50 cells was calculated. For the cell viability assay, 2×10^3^ cells were cultured in 96-well plates using the CCK8 assay kit to measure cell viability after the indicated treatments.

### Western blot analysis

The western blot assay was conducted as previously described 22. Briefly, cells were lysed with radioimmunoprecipitation assay (RIPA) buffer (Solarbio, Beijing, China) supplemented with 1% PMSF for 30 min on ice. Proteins were separated on SDS-PAGE, transferred onto PVDF membranes which were blocked with 5% BSA for 2 h at room temperature, followed by incubation with the following antibodies: bax (5032, CST, Boston, MA, USA, 1:1000), bcl-2 (15071, CST, Boston, MA, USA, 1:1000), caspase 3 (9668, CST, Boston, MA, USA, 1:1000), caspase 7 (9492, CST, Boston, MA, USA, 1:1000), β-tubulin (2146, CST, Boston, MA, USA, 1:10,000), γH2AX (ab81299, Abcam,1:1000), cyclin B (12231, CST, Boston, MA, USA, 1:1000), cdc2 (DF6024, afffinity, China, 1:1000), p-cdc2 (AF3108, afffinity, China, 1:1000, 1:1000), and GAPDH (Millipore, Billerica, MA, USA, 1:2000) at 4 °C for 12 h. Next, membranes were washed three times with phosphate buffered saline-Tween 20 (PBST) for 15 min and subsequently incubated with secondary antibodies (Promega, 1:10,000) for 1 h. Finally, images were detected with a gel imaging system (Syngene G: BOX Chemi XT4; Syngene; Cambridge, UK).

### RNA extraction and qRT-PCR

Total RNA was extracted using TRIzol reagent (Invitrogen). Lysates were mixed with chloroform and centrifuged at 12,000 g for 15 min at 4 °C. The upper aqueous phase was collected to a 1.5 ml tube, and an equal volume of isopropanol was added into it. cDNAs were synthesized using the Prime Script RT Kit (Promega; Madison, 498 WI, USA), according to the manufacturer's protocols. qRT-PCR analyses were performed using the SYBR Premix Ex Taq kit (Takara), employing GAPDH as an internal reference. The relative quantification values for RNAs were quantified using the 2^-ΔΔCt^ method. The prime sequences were listed in supplement Table S3.

### TEM and scanning electron microscopy (SEM)

For TEM, cells were fixed with 2.5% glutaraldehyde, followed by 1% osmium tetroxide treatment, and dehydration in graded ethanol, finally embedding the samples in epoxy resin. 70 nm ultra-thin slices were sectioned, stained with 2% uranyl acetate and lead citrate, and finally imaged with the H-7760 microscope. For SEM, cells were fixed with 2.5% glutaraldehyde at 4 °C and rinsed with 0.1 M phosphate buffered saline thrice. After fixing in 1% osmium tetroxide, these samples were dehydrated through an ascending ethanol gradient and dried with hexamethyldisilazane. Finally, the samples were sputtered with gold-palladium and observed under a scanning electron microscope (SEM, JSM-7900F; Japan).

### High-performance liquid chromatography (HPLC)

TMZ concentrations were measured through an HPLC system. The analytical column was ODS C18 column (250 mm × 4.6 mm). The mobile phase was composed of methanol containing 0.5% of acetic acid solution (1:9) at a flow rate of 1 mL/min. TMZ was detected at a wavelength of 329 nm. 10 µl of each sample was loaded on the HPLC column.

### LC-MS sample preparation

Plasma sample treatment: 50 µL plasma was added to 100 µL of 50% methanol aqueous solution containing 0.1% formic acid, then centrifuged at 15294 g for 10 min. The supernatant was collected, dried under N2, and resuspended in 100 µL of 50% methanol aqueous solution containing 0.1% formic acid. The mixture was vortexed for 30 s followed by centrifuging at 12,000 g for 10 min. Finally, 5 µL of the sample was injected into the LC-MS system for analysis.

Brain tissue: Animals were transcardially perfused with ice-cold saline, freezing immediately the brain and glioma tissues. These tissues were homogenized in a frozen grinding instrument at 4 °C, 65 Hz grinding speed for 4min, stopped for 5 s per minute. 100 µL sample was added to the mixture of 100 µL 50% methanol aqueous solution containing 0.1% formic acid and 50 µL internal standard fluid, and 1 mL of ethyl acetate containing 0.1% formic acid, then was centrifuged at 12,000 g for 10 min. The supernatant was collected, dried under N2, and resuspended in 100 µL 50% methanol aqueous solution containing 0.1% formic acid. Finally, 5 µL of supernatant was injected into the LC-MS system for analysis.

### Liquid chromatography coupled to triple-quadrupole mass spectrometry and conditions

Chromatographic separation was implemented on an Agilent eclipse plus C18 column (100×2.1 mm, 3.5 μm). The mobile phases consisted of 0.1% formic acid solution (phase A) and contained 0.1% formic acid-acetonitrile (phase B) at the flow rate of 0.4 mL/min. The samples were analyzed using the U3000 Liquid Chromatography (Thermo) and the API4000 Triple Quadrupole Mass Spectrum (AB SCIEX), using the following operation parameters: ion spray voltage (5000 V), curtain gas (25 psi), cover (5 psi), collision gas pressure (270 kpa), ion source temperature (500 °C), atomization gas (GSI) (50 psi), and auxiliary heating (GS2) (50 psi).

### Flow cytometry analysis

Cell cycle and cell apoptosis assays in glioma cells were carried out using the Cell Cycle and Apoptosis Analysis Kit (BestBio; Shanghai, China) after the indicated treatments. Finally, cells were analyzed using flow cytometry (BD FACSCanto II).

### *In vivo* intracranial patient-derived xenograft model

Animal experiments were performed according to the animal study protocols approved by the Institutional Animal Care and Use Committee at Tianjin Medical University. BALB/c nude mice were used to construct an intracranial orthotopic glioma model as previously reported 9. Cells were injected into the mouse brain under the guidance of a stereotactic instrument at coordinates relative to bregma: 2.0 mm posterior, 2.0 mm lateral, and 3.0 mm ventral. Bioluminescence imaging was performed to detect intracranial tumor growth on days 7, 14, and 21 through the In Vivo Imaging System (IVIS) Spectrum. Finally, mice were sacrificed and their brain tissues were removed. Mice brain tissues were fixed in 4% PFA for 24 h, embedded in paraffin, and sectioned into 5 μm slices. Kaplan-Meier survival curve was used to evaluate the animal's survival condition.

### H&E and immunohistochemical (IHC) staining

Brain sections were cut from paraffin-embedded brain tissue blocks. Brain sections were dewaxed, hydrated, and treated for antigen retrieval in citrate buffer at 100 °C for 20 min. Paraffin-embedded tissue sections were used for H&E staining. For IHC, tissue sections were first incubated with goat serum (Zhongshan Golden Bridge Bio-technology, Beijing, China) for 30 min at room temperature, then incubated with the primary antibody Ki-67 (9109, CST, 1: 200) and CD31 (ab28364, abcam, 1:50) at 4 °C for 12 h and subsequently incubated with HRP-conjugated secondary antibody (Zhongshan Golden Bridge Bio-technology, Beijing, China) for 1 h at room temperature. Sections were incubated with diaminobenzidine (DAB) (Zhongshan Golden Bridge Bio-technology, Beijing, China) followed by image acquisition with a microscope.

### Terminal deoxynucleotidyl transferase dUTP Nick End Labeling (TUNEL) Staining

Cultured cells were incubated with 50 μL of TUNEL detection solution (Beyotime, Beijing, China) at 37 °C for 1 h in the dark. Next, cells were stained with DAPI at 37 °C for 5 min in the dark. Finally, the cells were photographed using laser confocal microscopy (FV1200, Olympus, Tokyo, Japan).

### Immunofluorescence (IF) assay

Cells were washed with PBS two times, fixed with 4% PFA for 15 min, permeabilized with 0.5% Triton X-100 for 20 min, incubated with 10% BSA for 1 h at room temperature, and then stained with anti γH2AX antibody (ab81299, abcam, 1:250). O6-MetG adduct was stained with mouse anti-(O6-MetG) antibody EM-2-3 (1:500) 12 h at 4 °C. Finally, cells were incubated with appropriate secondary antibodies for 1 h, counter stained with DAPI (DAPI, CA, USA), and photographed with laser confocal microscope (FV1200, Olympus, Tokyo, Japan).

Brain sections were incubated with the following primary antibodies: rabbit polyclonal anti-γH2AX (ab81299, abcam, 1:250) and PTRF (18892-1-AP, proteintech, 1:200) overnight at 4 °C. Then, the brain sections were incubated with appropriate secondary antibodies, stained with DAPI (F6057, Sigma, USA), and photographed with laser confocal microscope (FV1200, Olympus, Tokyo, Japan).

### Statistical Analysis

All statistical analyses were performed with SPSS 22.0 software and GraphPad Prism 8 software. The student's *t*-test was used to compare two experimental groups, and one-way or two-way ANOVA was used to compare three or more experimental groups. The error bars in the figures represent the mean ± standard deviation (SD). Significance was defined as *p < 0.05, **p < 0.01, ***p < 0.001, ****p < 0.0001, ns = not significant. All results repeated at least three independent times.

## Results

### High PTRF expression confers a worse prognosis of GBM patients after TMZ treatment

**Figure 1:**
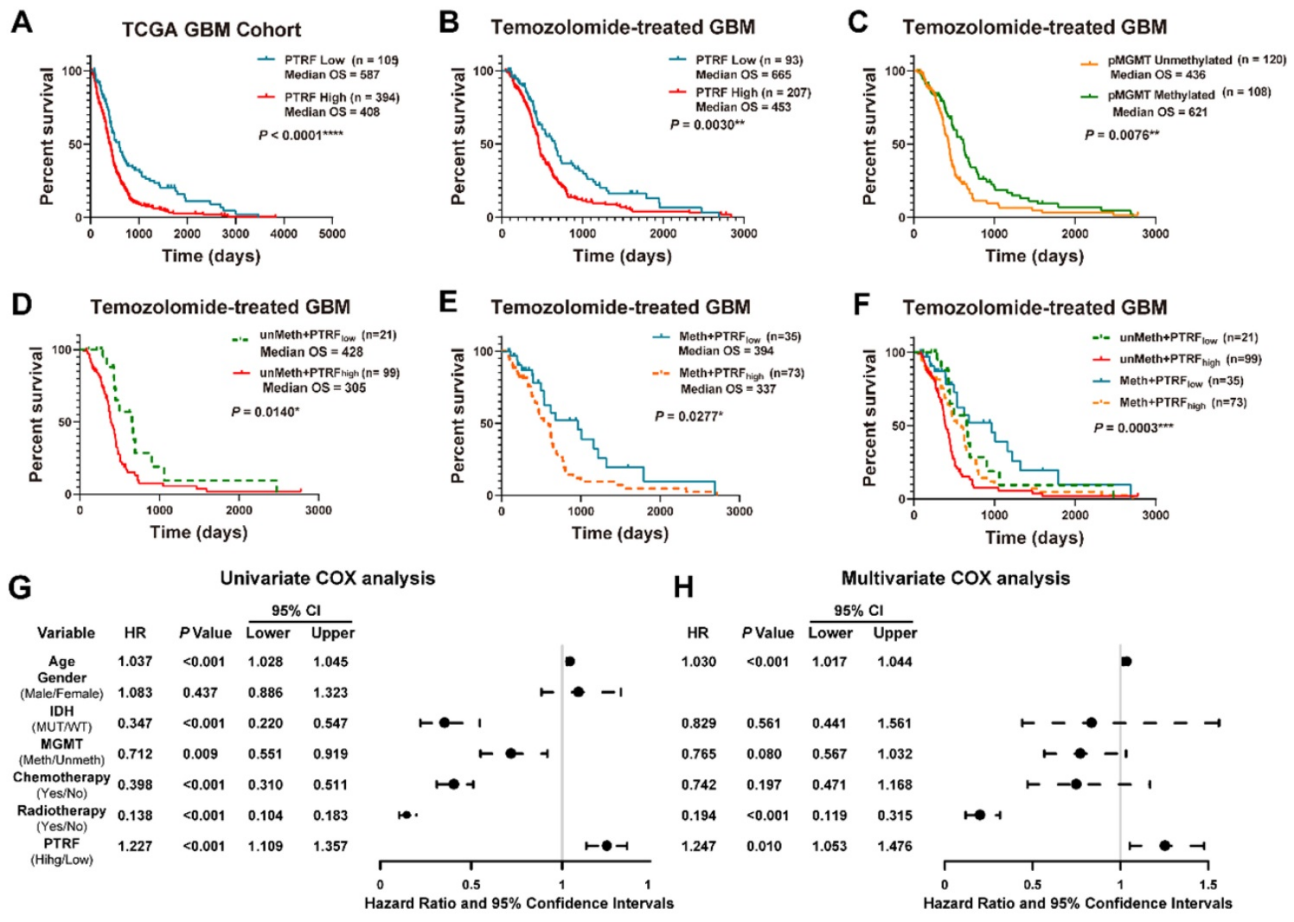
** High PTRF expression confers a worse prognosis of TMZ treatment in GBM patients. (A)** Kaplan-Meier survival analysis of the expression levels of PTRF in GBM patients based on the microarray data. **(B)** Kaplan-Meier survival analysis of the expression levels of PTRF in TMZ-treated GBM patients based on the microarray data. **(C)** Kaplan-Meier survival analysis of GBM patients with unmethylated or methylated pMGMT with TMZ treatment based on the microarray data. **(D-F)** Kaplan-Meier survival analysis of the effect of PTRF expression level on MGMT unmethylated or methylated TMZ-treated GBM patients. **(G-H)** Univariate and multivariate analyses of the PTRF expression and other clinical information in relation to the overall survival in the TCGA GBM cohort.

Studies have shown PTRF potential as a promising biomarker in the prognosis of GBM in the clinical setting 23. Indeed, the prognosis of GBM patients was negatively correlated with PTRF expression (Figure 1A and Figure S1A). Previous study had reported PTRF might act as a positive modulator in multidrug resistant (MDR) of GBM patients and could modulate the sensitivity of GBM cells to some anticancer drugs 10. Since there are no reports on PTRF role in TMZ-resistance, we sought to investigate the implication of PTRF expression in developing TMZ chemoresistance. The analyses of GBM gene expression profiles from the TCGA and CGGA databases show that the prognosis of GBM patients treated with TMZ is negatively correlated with PTRF expression (Figure 1B and Figure S1B). The effect of PTRF on the prognosis of TMZ-treated GBM patients is independent of the methylation of O^6^-Methylguanine-DNA Methyltransferase (MGMT) promoter (pMGMT) (Figure 1C-F and Figure S1C-F). Moreover, univariate Cox regression analysis from the CGGA and TCGA cohorts suggested that high expression of PTRF, older age, IDH1 gene mutation, chemotherapy, and radiotherapy were associated with overall survival outcomes (Figure 1G and Figure S1G). Further analysis using the multivariate Cox regression analysis revealed that PTRF expression was correlated with overall survival (Figure 1H and Figure S1H). Therefore, PTRF can act as a new biomarker independent of MGMT expression in predicting the prognosis of GBM patients after TMZ treatment.

### PTRF increases TMZ resistance in GBM

**Figure 2:**
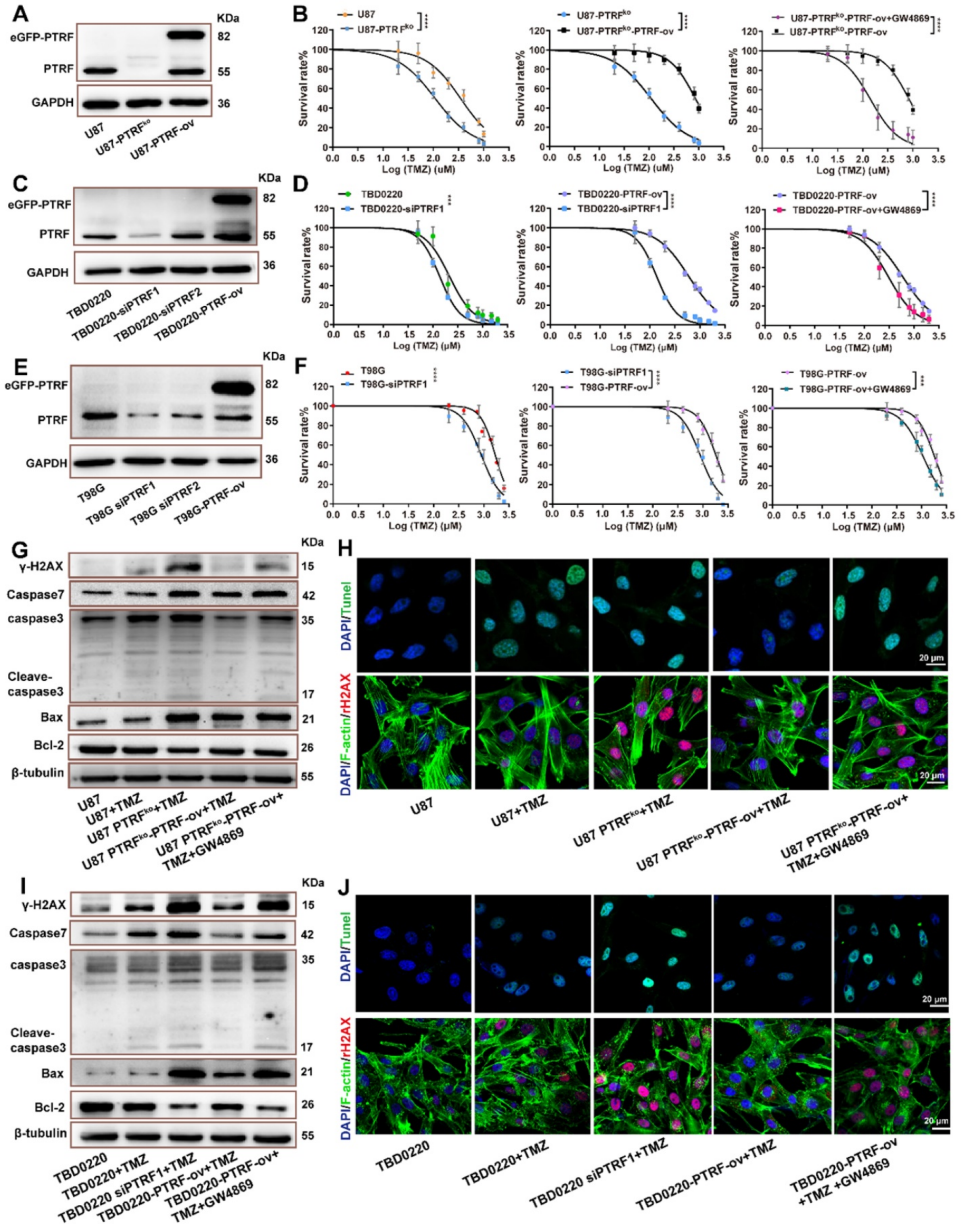
** PTRF enhances TMZ resistance. (A)** Representative western blot images showing the expression of PTRF in U87, U87-PTRF-ov, and U87 PTRF^ko^ groups. **(B)** The cell survival rate was measured after TMZ treatment in U87, U87-PTRF^ko^, U87-PTRF^ko^-PTRF-ov, and U87-PTRF^ko^-PTRF-ov+GW4869 groups. Data are represented as the mean ± SEM (n = 6). ****p < 0.0001. **(C)** Representative western blotting showing the expression of PTRF in TBD0220, TBD0220-siPTRF1 and TBD0220-PTRF-ov groups. **(D)** The cell survival rate was measured after TMZ treatment in TBD0220, TBD0220-siPTRF1, TBD0220-PTRF-ov, and TBD0220-PTRF-ov+GW4869 groups. Data are represented as the mean ± SEM (n = 6). ****p < 0.0001. **(E)** Representative western blotting showing the expression of PTRF in T98G, T98G-siPTRF and T98G-PTRF-ov groups. **(F)** The cell survival rate was measured after TMZ treatment in T98G, T98G-siPTRF1, T98G-PTRF-ov, and T98G-PTRF-ov+GW4869 groups. Data are represented as the mean ± SEM (n = 6). ***p < 0.001, ****p < 0.0001. **(G)** Western blot analysis showing the protein expression of bax, bcl-2, caspase 3, caspase 7, and γ-H2AX after TMZ treatment in U87, U87-PTRF^ko^, U87-PTRF^ko^-PTRF-ov, and U87-PTRF^ko^-PTRF-ov+GW4869 groups. **(H)** IF showing the population of TUNEL and γ-H2AX positive cells after TMZ treatment in U87, U87-PTRF^ko^, U87-PTRF^ko^-PTRF-ov, and U87-PTRF^ko^-PTRF-ov+GW4869 groups. Scale bar = 20 μm.** (I)** Representative western blotting showing the protein expression of bax, bcl-2, caspase 3, caspase 7, and γ-H2AX after TMZ treatment in TBD0220, TBD0220-siPTRF1, TBD0220-PTRF-ov, and TBD0220-PTRF-ov+GW4869 groups. **(J)** IF showing the population of TUNEL and γ-H2AX positive cells after TMZ treatment in TBD0220, TBD0220-siPTRF1, TBD0220-PTRF-ov, and TBD0220-PTRF-ov+GW4869 groups. Scale bar = 20 μm.

A positive correlation between EV shedding-related genes and drug resistance in cancer cells has been described 23. Thus, it is possible that the function of PTRF on EXO secretion might be involved in developing TMZ resistance in glioma. First, cell lines with PTRF overexpression or knockdown/knockout were established to verify the expression levels by western blotting (Figure 2A, C, E and Figure S2D-F) and qRT-PCR (Figure S2B-C). T98G, U87, and TBD0220 cell lines were selected to evaluate whether the function of PTRF on the efficacy of TMZ in GBM was associated with MGMT expression. MGMT mRNA expression levels were high in T98G, and low in U87/TBD0220 cells (Figure S2A).

U87, TBD0220 and T98G cell lines were exposed to increasing TMZ concentrations yielding dose-dependent proliferation inhibition after 48 hours of treatment. U87, U87-PTRF^ko^, U87-PTRF^ko^-PTRF-ov, and U87-PTRF^ko^-PTRF-ov+GW4869 (an inhibitor of exosome biogenesis/release) cells were exposed to increasing TMZ concentrations. We found the IC50 of TMZ decreased in U87-PTRF^ko^ compared to U87 group, and increased in U87-PTRF^ko^-PTRF-ov group compared to U87-PTRF^ko^ group, but decreased after treated with 20 µM GW4869 (Figure 2B and supplement Table S4). Cell colony formation assays were performed in U87, U87-PTRF^ko^, U87-PTRF^ko^-PTRF-ov, and U87-PTRF^ko^-PTRF-ov+GW4869 under the treatment of 200 µM TMZ. The results revealed that the number of clones was decreased in U87-PTRF^ko^ group compared to U87 group, and increased in U87-PTRF^ko^-PTRF-ov group compared to U87-PTRF^ko^ group after TMZ treatment; however, the addition of GW4869 reversed these effects (Figure S3A). Similarly, TBD0220 and T98G cells were exposed to increasing TMZ concentrations. We found the IC50 value of TMZ decreased in TBD0220-and T98G-siPTRF group compared to TBD0220-and T98G group, and increased in TBD0220-and T98G-PTRF-ov group compared to TBD0220-and T98G-siPTRF group, but decreased after treated with 20- μM GW4869 (Figure 2D,F and supplement Table S5,6). Cell colony formation assays were also performed in TBD0220-and T98G, TBD0220-and T98G-siPTRF, TBD0220-and T98G-PTRF-ov and TBD0220-and T98G-PTRF-ov+GW4869 groups; TBD0220 and T98G lines were treated with 200 µM and 800 µM of TMZ, respectively. The results also showed decreased cell proliferation in siPTRF1+TMZ group when compared to TMZ group, but increased in PTRF-ov+TMZ group compared to siPTRF1+TMZ group; addition of GW4869 decreased this effect (Figure S3B, C). Furthermore, western blot measured the expression of bax, bcl-2, caspase 3, caspase 7, γH2AX in U87, TBD0220 and T98G lines after the indicated treatments. TMZ treatment increased the expressions of DNA damage and apoptosis related proteins in PTRF knockdown/knockout group, while TMZ treatment exerted protective effects inhibiting the expression of these proteins in PTRF-ov group. Interestingly, these protective effects in PTRF-ov cells were reversed by GW4869 (Figure 2G, I and Figure S3D). IF analysis measured the level of TUNEL and γH2AX positive U87 and TBD0220 cells, demonstrating that apoptosis and DNA damage were enhanced in the PTRF knockout/knock down group, unlike the PTRF-ov group; further, the addition of GW4869 reversed the expression profile (Figure 2H, J). Likewise, IF analysis of γH2AX in T98G cell lines revealed that the PTRF knockdown increased genomic instability, while PTRF-ov had a protective role in preventing genome damage, but addition of GW4869 in these cells elevated the DNA damage levels (Figure S3E).

### PTRF knockout increases intracellular TMZ concentration by decreasing EVs-mediated TMZ efflux

**Figure 3:**
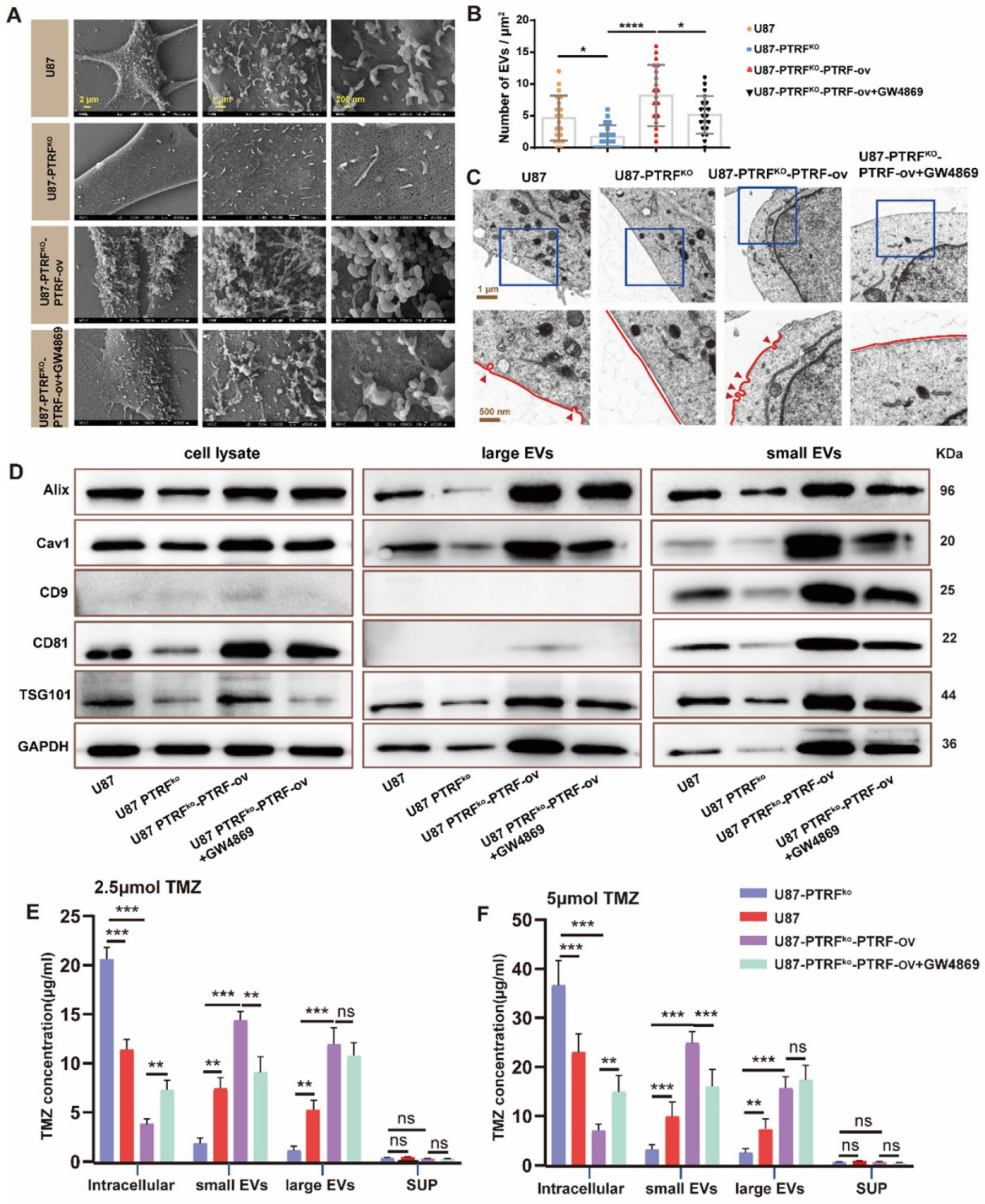
** PTRF knockout increases intracellular TMZ concentration by decreasing the production of EVs. (A)** SEM analysis of EVs produced from U87 cells. **(B)** The EV number was calculated in U87 cells. Data are represented as the mean ± SEM (n = 3). *p < 0.05, ****p < 0.0001. **(C)** TEM analysis of caveola in U87 cells. Red arrowheads represent caveolae. **(D)** Protein expression levels of Alix, CD81, CD9, cav1, TSG101 and GAPDH in EVs and cell lysate.** (E)** TMZ concentration in intracellular, small EVs, large EVs, and supernatant was analyzed by HPLC after 2.5 μmol TMZ treatment. Data are represented as the mean ± SEM (n = 3). **p < 0.01, ***p < 0.001, ns represents *p* > 0.05.** (F)** TMZ concentration in intracellular, small EVs, large EVs, and supernatant was analyzed by HPLC after 5 μmol TMZ treatment. Data are represented as the mean ± SEM (n = 3). **p < 0.01, ***p < 0.001, ns represents *p* > 0.05.

To examine whether the effect of PTRF on TMZ-resistance is mediated through EVs secretion, EVs and the caveolae were evaluated with SEM and TEM, respectively, in U87, U87-PTRF^ko^, U87-PTRF^ko^-PTRF-ov and U87-PTRF^ko^-PTRF-ov+GW4869 cells. The numbers of EVs and the caveolae were positively correlated to PTRF expression, and decreasing these numbers after GW4869 treatment (Figure 3A-C). Caveolin expression is also correlated to PTRF levels, being this reduced after GW4869 treatment (Figure S4A). The typical morphology and size of EVs in different groups were determined using TEM and NanoSight system. These results indicated the expression level of PTRF and GW4869 treatment did not influence the morphology and size of EVs (Figure S4B, C, D). The small and large EVs were extracted from the same number of different treatment cells. Small and large EVs concentration decreased or increased after knockout or overexpression of PTRF, respectively, small EVs with a partial decrease after GW4869 treatment but large EVs did not decrease (Figure S4E, F, G, H). Consistent with the quantification of EVs, the expressions of Alix, CD81, CAV1, CD9, and TSG101 were decreased in U87-PTRF^ko^ EVs, increased in U87-PTRF^ko^-PTRF-ov EVs, and further decreased in U87-PTRF^ko^-PTRF-ov+GW4869 EVs (Figure 3D). The protein expression of CD9, Alix, Tsg101 and CD81 were also measured in the cell lysates (Figure 3D). The expressions of Alix, CD81, CAV1, CD9, and TSG101 were decreased in the cell lysates of U87-PTRF^ko^ group, increased in U87-PTRF^ko^-PTRF-ov cell lysates, and only CD81, CAV1, CD9, TSG101 further decreased in U87-PTRF^ko^-PTRF+GW4869 group. Subsequently, TMZ concentrations were measured in cells, small EVs, large EVs as well as in the supernatant of all groups after being treated with 2.5 or 5 µmol of TMZ. The results revealed the increased intracellular and decreased small and large EVs TMZ concentration in the U87-PTRF^ko^ group compared to U87 group, while the opposite effect was observed in the U87-PTRF^ko^-PTRF-ov group compared to U87-PTRF^ko^ group, and intracellular and small EVs TMZ concentration was further reversed by GW4869 treatment but not include large EVs (Figure 3E-F and Figure S5A-B). This phenomenon may be explained by the function of GW4869, which mainly inhibit exosome (< 200 nm) biogenesis/release but not inhibit large EVs 24. Together, these results suggest that PTRF modulates intracellular TMZ concentration by increasing TMZ efflux mediated by EVs.

### PTRF knockout enhances the efficacy of TMZ in orthotopic xenograft glioma mice

**Figure 4:**
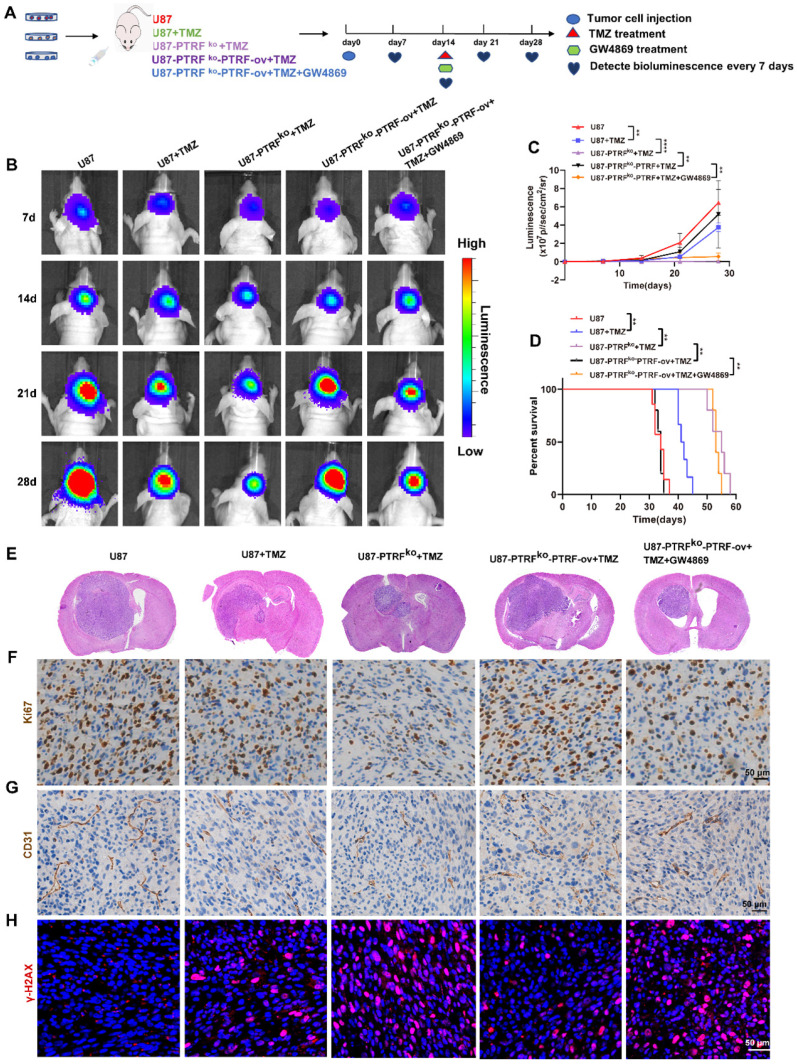
** PTRF knockout enhances the efficacy of TMZ in orthotopic xenograft glioma mice. (A)** Schematic illustration of the GBM orthotopic xenograft model. **(B)** Bioluminescence images of tumor growth after tumor implantation. n = 5-7 for each group. **(C)** Tumor growth curves by quantification of bioluminescent imaging signal intensities. Data are represented as the mean ± SEM (n = 5-7). **** *p* < 0.0001.** (D)** Kaplan-Meier survival curve of nude mice. Data are represented as the mean ± SEM (n = 5-7). **p < 0.01. **(E)** Representative images of H&E staining showing tumor volume in the nude mice. **(F)** IHC staining for Ki67 in brain tumor samples. Scale bar = 50 μm. **(G)** IHC of CD31 expression in the brain tumor. Scale bar = 50 μm. **(H)** IF of γH2AX expression in the brain tumor. Scale bar = 50 μm.

To further investigate the function of PTRF on the efficacy of TMZ *in vivo*, an orthotopic GBM mice model was generated by intracranially injecting three groups of cells separately into mice brains. Then mice were intraperitoneally injected with DMSO, TMZ (5 mg/kg/day) or GW4869 (2 mg/kg/day) at the frequency of 5 days ON and 2 days OFF for 2 weeks (Figure 4A). The bioluminescence imaging shows that those tumors established with U87-PTRF^ko^ cells were more sensitive to TMZ treatment when compared to U87-PTRF^ko^-PTRF-ov tumors, whereas U87-PTRF^ko^-PTRF-ov tumors treated with GW4869 recovered sensitivity to TMZ (Figure 4B-C). Kaplan-Meier survival curves indicated that the median survival time of the mice was 34 days in U87 group, 41 days in U87+TMZ group, 55 days in U87-PTRF^ko^+TMZ group, 34 days in U87-PTRF^ko^-PTRF-ov+TMZ; GW4869 treatment prolonged the median survival time to 53 days (Figure 4D). Furthermore, H&E staining confirmed the smaller size of tumors in U87-PTRF^ko^+TMZ group compared to U87+TMZ group; in addition, it revealed a significant increase of tumor burden in the U87-PTRF^ko^-PTRF-ov+TMZ group compared to U87-PTRF^ko^+TMZ group, while GW4869 treatment decreased tumor size (Figure 4E). Ki67 staining also suggested a lower percentage of proliferating cells in U87-PTRF^ko^+TMZ group compared to U87+TMZ group and revealed a modest inhibition of cell proliferation in U87-PTRF^ko^-PTRF-ov+TMZ group; the addition of GW4869 significantly decreased the cell proliferation rate (Figure 4F). Considering the effects of vascularization on GBM, we observed the number of the microvessels by staining CD31. The results suggested the number of the microvessels decreased in U87-PTRF^ko^+TMZ group compared to U87+TMZ group, increased in U87-PTRF^ko^-PTRF-ov+TMZ group compared to U87-PTRF^ko^+TMZ group; GW4869 treatment significantly reversed this change (Figure 4G). To further verify these results, the expression of γH2AX was measured, demonstrating an increased expression in U87-PTRF^ko^+TMZ group compared to U87+TMZ group, being this lower in U87-PTRF^ko^-PTRF-ov+TMZ group in contrast to U87-PTRF^ko^+TMZ group; GW4869 treatment significantly reversed γH2AX expression (Figure 4H).

### Sequential therapy of TMZ followed by CQ decreases TMZ resistance

**Figure 5:**
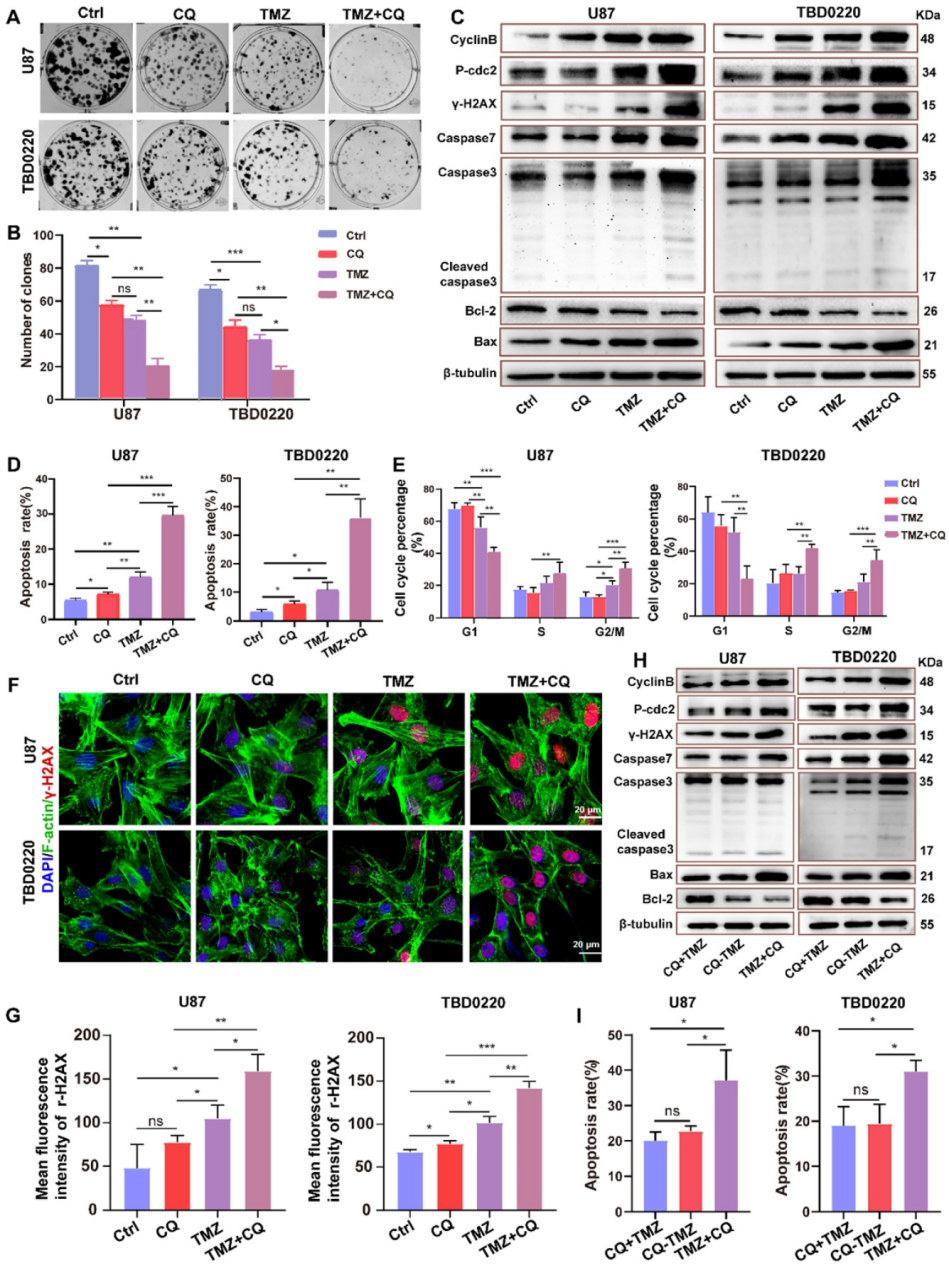
** Sequential therapy of TMZ plus CQ promotes TMZ efficacy by increasing intracellular TMZ concentration. (A-B)** A colony formation assay was performed in GBM cells. Data are represented as the mean ± SEM (n = 3). *p < 0.05, **p < 0.01, ***p < 0.001, ns represents *p* > 0.05. **(C)** Western blot analysis showing the protein expression of bax, bcl-2, caspase 3, caspase 7, cyclin B, p-cdc2, and γH2AX after TMZ and/or CQ treatment in GBM cells. **(D)** The apoptosis rate in GBM cells after TMZ and/or CQ treatment. Data are represented as the mean ± SEM (n = 3). *p < 0.05, **p < 0.01, ***p < 0.001. **(E)** Cell cycle distribution after exposure to TMZ and/or CQ in GBM cells. Data are represented as the mean ± SEM (n = 3). *p < 0.05, **p < 0.01, ***p < 0.001. **(F-G)** IF showing the population of γH2AX positive cells after treating with TMZ or CQ in GBM cells. Data are represented as the mean ± SEM (n = 3). *p < 0.05, **p < 0.01, ***p < 0.001, ns represents *p* > 0.05. **(H)** Protein levels of bax, bcl-2, caspase 3, caspase 7, γH2AX, p-cdc2 and cyclinB in CQ+TMZ, CQ-TMZ, and TMZ+CQ treatment groups.** (I)** Apoptosis rate in CQ+TMZ, CQ-TMZ, and TMZ+CQ treatment groups. Data are represented as the mean ± SEM (n = 3). *p < 0.05, ns represents *p* > 0.05.

In order to better achieve clinical transformation, based on the above findings and the fact that CQ can effectively alleviate multidrug resistance of cancer cells through preventing and delaying the outward transport of antineoplastic drugs 17, we hypothesized that CQ might enhance TMZ efficacy by increasing intracellular TMZ concentration. First, a colony formation assay was performed in U87 and TBD0220 lines under the treatment regimen of 25 µM CQ, 200 µM TMZ and TMZ +CQ (25 µM CQ was added after 8 h of 200 µM TMZ treatment). Colon numbers were significantly reduced in U87-and TBD0220-TMZ+CQ groups compared to those treated with TMZ or CQ alone (Figure 5A-B). Also, the combination of TMZ and CQ upregulated the levels of bax, caspase 3, caspase 7, and γH2AX compared to either agent alone (Figure 5C). In addition, the apoptosis rate of GBM cells elevated in the TMZ+CQ treatment group (Figure 5D and Figure S6A). Western blotting revealed higher protein expression of cyclin B and p-cdc2 in TMZ+CQ groups compared to TMZ or CQ monotreatment (Figure 5C). Consistent with these findings, flow cytometry also revealed a significant increase in the percentage of G2/M cells in TMZ+CQ groups (Figure 5E and Figure S6B). IF analysis indicated increased expression of γH2AX in TMZ+CQ groups compared to the mono treatments (Figure 5F-G). Furthermore, considering the function of CQ in inhibiting endocytosis, we tested whether the efficacy of sequential therapy of TMZ followed by CQ was more efficient compared to CQ+TMZ (200 µM TMZ was added after 25 µM CQ treatment for 8 h) and CQ-TMZ groups (25 µM CQ and 200 µM TMZ were treated at the same time). It was demonstrated that the expression levels of apoptosis and DNA damage response related proteins were higher in U87-and TBD0220 TMZ+CQ groups compared to those in CQ+TMZ and CQ-TMZ groups (Figure 5H). Western blotting also revealed higher protein expression of cyclin B and p-cdc2 in TMZ+CQ group compared to CQ+TMZ and CQ-TMZ groups (Figure 5H). Consistent with these findings, flow cytometry analysis also showed that the apoptosis rate was elevated in TMZ+CQ group compared to CQ+TMZ and CQ-TMZ (Figure 5I and Figure S6C).

### Sequential therapy of TMZ followed by CQ in orthotopic xenograft glioma mice

**Figure 6:**
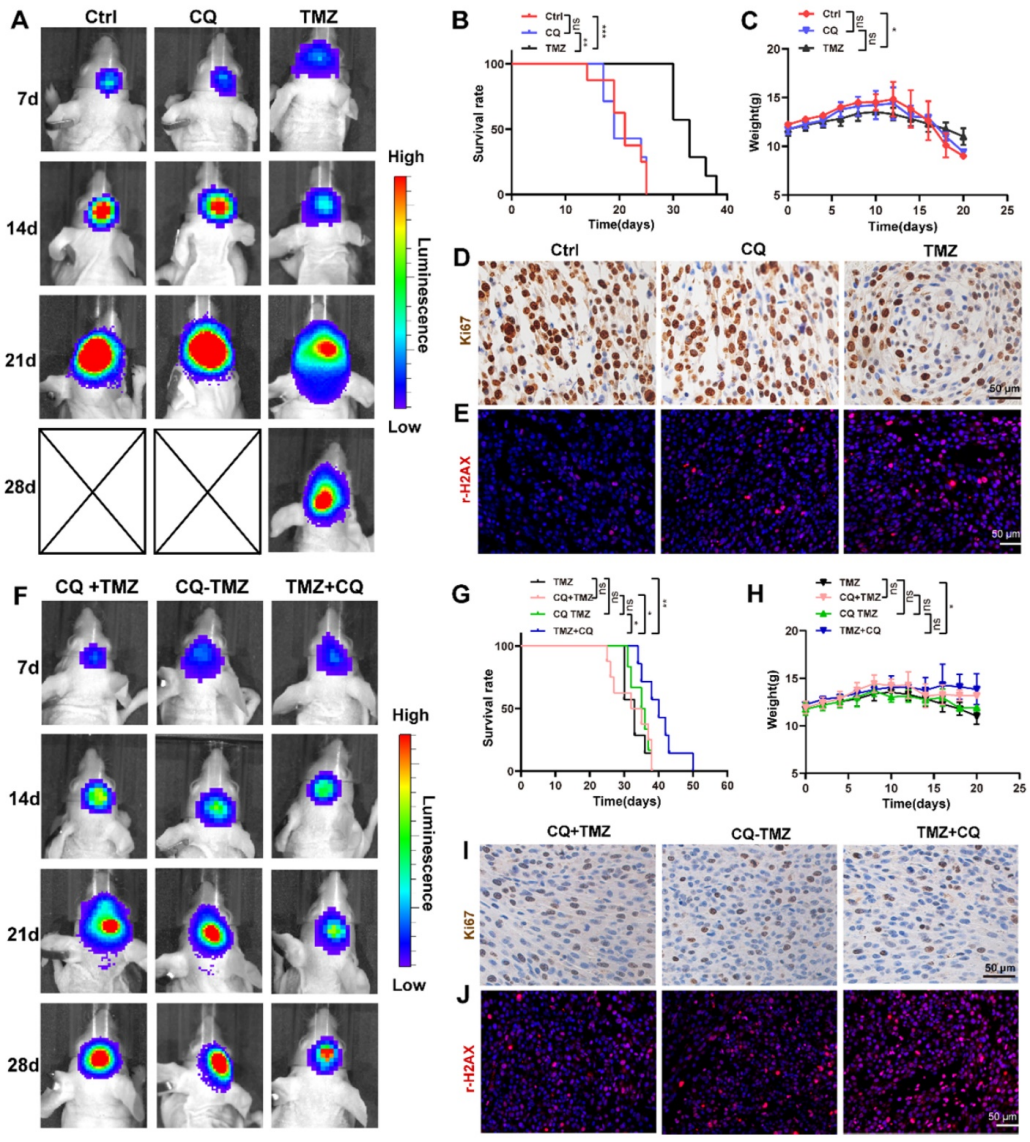
** Sequential therapy of TMZ followed by CQ in orthotopic xenograft glioma mice. (A)** ude mice after treating with TMZ or CQ. Data are represented as the mean ± SEM (n = 6-7). **p < 0.01, ***p < 0.001, ns represents *p* > 0.05. **(C)** Bodyweight of mice after TMZ or CQ treatment over time. Data are represented as the mean ± SEM (n = 6-7). *p < 0.05, ns represents *p* > 0.05. **(D)** IHC staining for Ki67 in brain tumor samples following TMZ or CQ treatment. Scale bar = 50 μm. **(E)** IF of γH2AX expression in the brain tumor after treating with TMZ or CQ. Scale bar = 50 μm. **(F)** Bioluminescence images of tumor growth in CQ+TMZ, CQ-TMZ, and TMZ+CQ groups after tumor implantation. **(G)** Kaplan-Meier survival curve in CQ+TMZ, CQ-TMZ, and TMZ+CQ groups of nude mice. Data are represented as the mean ± SEM (n = 6-7). *p < 0.05, **p < 0.01, ns represents *p* > 0.05. **(H)** Bodyweight of mice in CQ+TMZ, CQ-TMZ, and TMZ+CQ groups. Data are represented as the mean ± SEM (n = 6). *p < 0.05, ns represents *p* > 0.05. **(I)** IHC staining of brain tumor samples for Ki67 in CQ+TMZ, CQ-TMZ, and TMZ+CQ groups. Scale bar = 50 μm. **(J)** IF of γH2AX expression in brain tumors in CQ+TMZ, CQ-TMZ, and TMZ+CQ groups. Scale bar = 50 μm.

To investigate whether sequential therapy of TMZ followed by CQ enhances TMZ efficacy *in vivo*, an orthotopic GBM mice model was established by intracranially injecting TBD0220 cells into mice brains. The mice were divided into six groups based on their treatments. Mice in CQ group were given CQ (20 mg/kg/day for 3 days every week for 2 weeks p.o.), and TMZ group was given TMZ (5 mg/kg/d for 5 days every week for 2 weeks p.o.). The bioluminescence imaging showed that TMZ treatment decreased the size of tumors compared to the control group, unlike CQ treatment (Figure 6A). Results also indicate that TMZ treatment enhanced the survival time and mice bodyweight while it reduced the cell proliferation rate compared to control and CQ groups (Figure 6B-D), with a higher expression of γH2AX (Figure 6E and Figure S7A).

To further demonstrate the efficacy of sequential treatment against glioma *in vivo*, mice were divided into three groups. CQ+TMZ group received CQ (20 mg/kg/day for 3 days every week for 2 weeks p.o.) and TMZ (from the third day given 5 mg/kg/day for 5 days every week for 2 weeks p.o.), CQ-TMZ group was treated with CQ (20 mg/kg/day for 3 days every week for 2 weeks p.o.) and TMZ (5 mg/kg/day for 5 days every week for 2 weeks p.o.), and TMZ+CQ group was given TMZ (5 mg/kg/day for 5 days every week for 2 weeks p.o.) and CQ (20 mg/kg/day from the fifth day for 3 days every week for 2 weeks p.o.). Bioluminescence imaging shows that TMZ+CQ group had the most potent effect in tumor volume reduction and median survival enhancement (Figure 6F-G); TMZ+CQ group prolonged the median survival time to 43 days compared to those in the TMZ (33 days), CQ+TMZ (33 days) and CQ-TMZ (35 days) groups. Interestingly, TMZ+CQ group did not show any increase in the bodyweight when compared to CQ+TMZ and CQ-TMZ groups (Figure 6H). Moreover, Ki67 staining also suggested a lower percentage of proliferating cells in the TMZ+CQ group in contrast to other treatment groups (Figure 6I), with a higher expression of γH2AX (Figure 6J and Figure S7A). Considering the effects of vascularization on GBM, we observed the number of the microvessels by staining CD31. The results suggested TMZ treatment decreased the number of the microvessels compared to ctrl group, and TMZ+CQ group decreased the number of the microvessels compared to TMZ, CQ+TMZ and CQ-TMZ groups (Figure S7B, C).

### Sequential therapy of TMZ followed by CQ decreases TMZ resistance by increasing intracellular TMZ concentration

**Figure 7:**
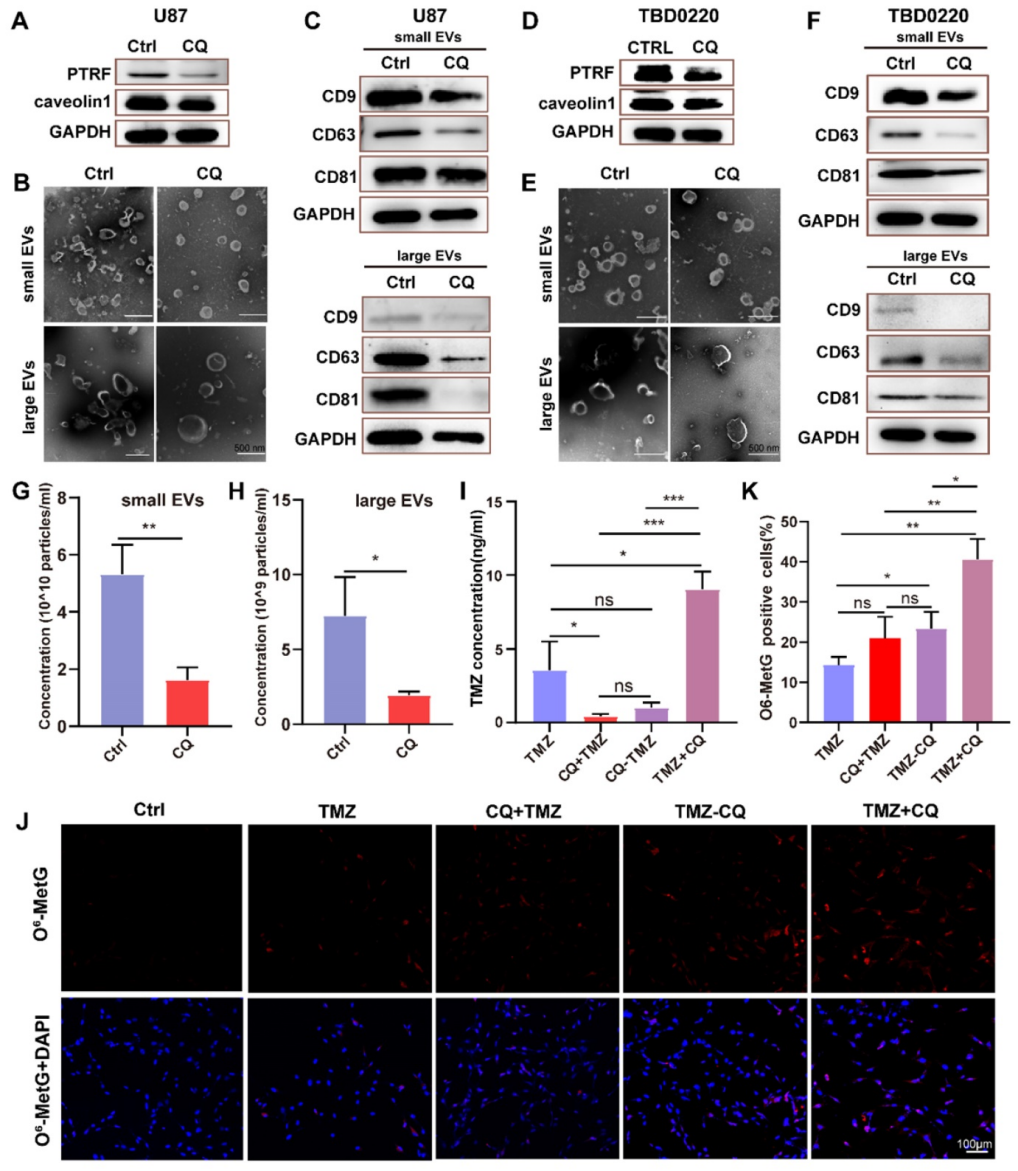
** Sequential therapy of TMZ plus CQ increases intracellular TMZ concentration. (A)** The expression of PTRF and caveolin1 were analyzed in CQ-treated U87 cells. **(B)** The morphology of small EVs and large EVs in CQ-treated U87 cells was observed by TEM. **(C)** Protein levels of CD63, CD81, and CD9 analyzed from small EVs and large EVs in CQ-treated U87 cells. **(D)** The expressions of PTRF and caveolin1 were analyzed in CQ-treated TBD0220 cells. **(E)** The morphology of small EVs and large EVs in CQ-treated TBD0220 cells was observed by TEM. **(F)** Protein expression levels of CD63, CD81, and CD9 were analyzed from small EVs and large EVs in CQ-treated TBD0220 cells.** (G-H)** The concentration of small EVs and large EVs in CQ-treated U87 cells. Data are represented as the mean ± SEM (n = 3). *p < 0.05, **p < 0.01.** (I)** The intracellular TMZ concentration was measured by LC-MS in TMZ, CQ+TMZ, CQ-TMZ, and TMZ+CQ treatment groups. Data are represented as the mean ± SEM (n = 3). *p < 0.05, ***p < 0.001, ns represents *p* > 0.05.** (J-K)** IF showing the population of O^6^-MetG expression in Ctrl, TMZ, CQ+TMZ, CQ-TMZ, and TMZ+CQ treatment groups in U87 cells. Data are represented as the mean ± SEM (n = 3). *p < 0.05, **p < 0.01, ns represents *p* > 0.05.

**Figure 8:**
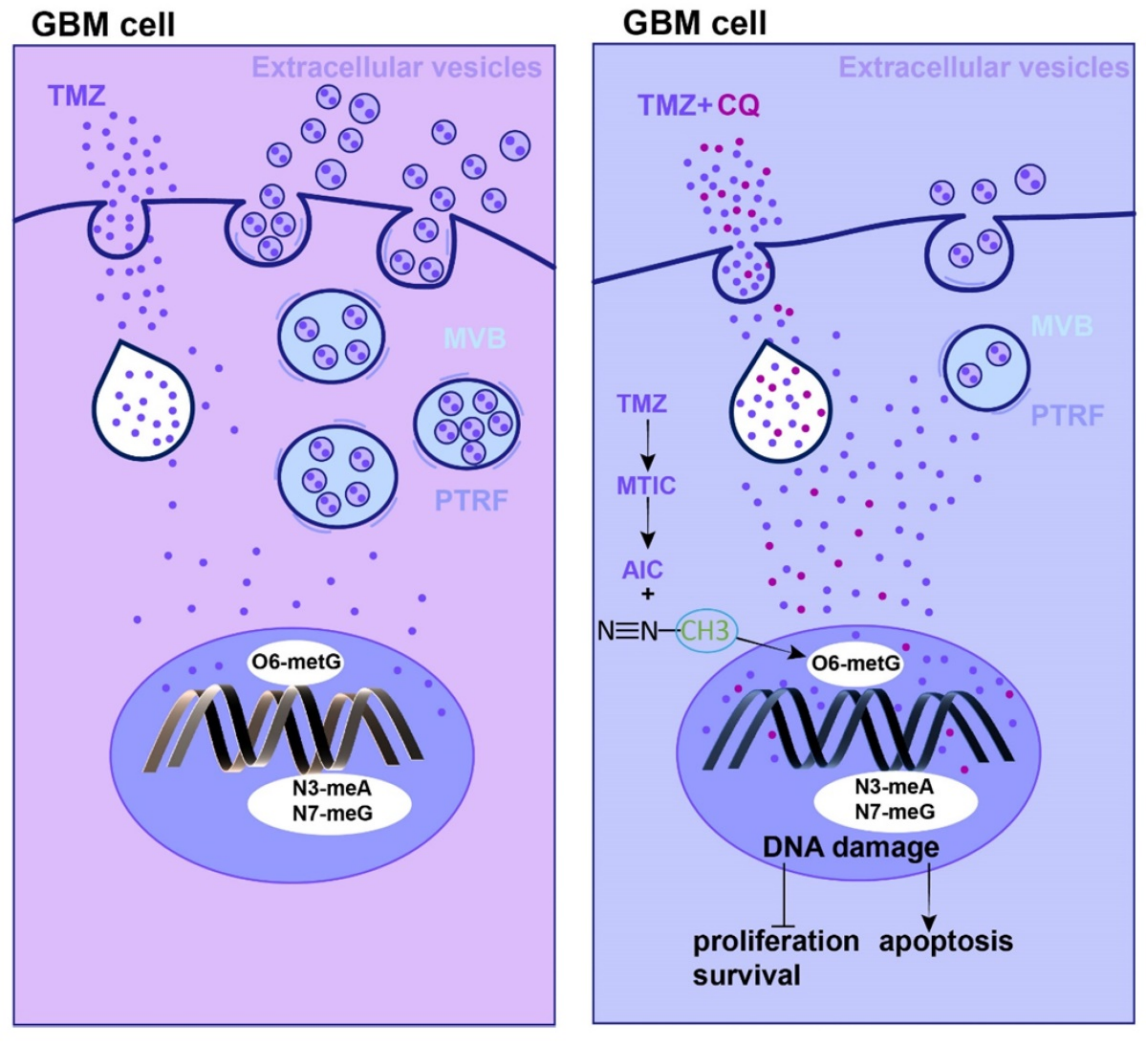
The mechanism of TMZ resistance and sequential therapy of TMZ followed by CQ for decreasing TMZ resistance in glioblastoma.

To verify whether CQ could enhance TMZ efficacy by increasing intracellular TMZ concentration, the caveolae, endocytic vesicles, and multivesicular body (MVB) formation was evaluated in U87 cells by TEM. As expected, there were fewer caveolae, endocytosis vesicles, and MVB in 25 µM CQ-treated cells compared to the control group (Figure S8A). As previous studies reported, PTRF expression was closely associated with the formation and secretion of the caveolae and EVs 5, 6. Therefore, the changes in the number of caveolae, vesicles, and MVBs caused by CQ treatment might have been mediated by PTRF. In fact, CQ treatment decreased the expression of PTRF and caveolin1 in U87 and TBD0220 cells, which are closely associated with the formation and secretion of EVs 5, 6 (Figure 7A, D). CQ treatment also decreased the expression of PTRF in vivo (Figure S8C). Next, the morphology and size of large EVs and small EVs were measured in CQ and control groups. We found CQ treatment did not change the morphology and size of large EVs and small EVs (Figure 7B, E). Consistent with the above results, protein levels of CD81, CD9, and CD63 downregulated in both large EVs and small EVs from U87 and TBD0220-CQ groups compared to the control (Figure 7C, F). Also, the concentration of particles was reduced in U87-CQ treatment group (Figure 7G-H). Consequently, we determined the intracellular TMZ concentration in TMZ, CQ+TMZ, CQ-TMZ, and TMZ+CQ treatment groups. Results revealed that the intracellular TMZ concentration was significantly enhanced in the TMZ+CQ group compared to those obtained in TMZ, CQ+TMZ, and CQ-TMZ groups (Figure 7I). IF analysis further confirm these results by measuring the levels of O^6^-Methyl-2-Deoxyguanosine (O^6^-MetG) in Ctrl, TMZ, CQ+TMZ, CQ-TMZ, and TMZ+CQ groups in U87 cells. These results suggested that TMZ+CQ treatment increased the expression of O^6^-MetG compared to the other treatment groups (Figure 7J-K). To this end, sequential therapy of TMZ followed by CQ may promote TMZ efficacy by increasing intracellular TMZ concentration (Figure 8). To evaluate the biosafety of repeated administrations of TMZ or CQ, at the end of experiment, three mice from each group were used for the study. We have analyzed the blood routine, liver kidney function and HE staining of the main organs (heart, liver, spleen, lung and kidney) of mice bearing orthotopic glioma after repeated administrations of TMZ and CQ (Table S7,8 and Figure S9). These results indicate that repeated administrations of TMZ and CQ have no obvious organ toxicity and other side effects on cells of normal tissues.

## Discussion

It has been shown that chemotherapy after surgery is the most effective treatment strategy against GBM. TMZ administration significantly prolongs the median survival time, but GBM patients gradually exhibit TMZ-resistance during the course of the treatment 3. In the past decades, many chemoresistance mechanisms have been reported, such as the expression of the DNA repair protein MGMT, drug efflux transporters, gap junction activity, DNA mismatch repair, DNA base excision repair, poly (ADP)-ribose polymerase repair system, the presence of glioma stem cells, upregulation of cell survival autophagy, the DNA double strand break repair, and the up-regulation of components of the ubiquitin proteasome system with oncogenic activity 25-31. In glioma, PTRF alters the tumor microenvironment by increasing exosome secretion, being associated with cell growth and promoting the immune response 5, 32, 33. GBM patients showed higher PTRF expression levels when compared to healthy and low-grade glioma subjects; PTRF levels in relapsed GBM patients were significantly higher than primary GBM patients 5, 10. In our study, we found that the prognosis of glioma patients treated with TMZ in the high-PTRF group was worse compared to those in the low-PTRF group. We also revealed that the prognosis of pMGMT methylated glioma patients treated with TMZ in the high-PTRF expression group was worse than those in low-PTRF expression. Therefore, we hypothesized that there might be an alternative mechanism mediated by PTRF in a MGMT-independent manner contributing to the TMZ resistance.

Although the biological functions of PTRF are well studied, the effect of PTRF on TMZ resistance is yet unknown. We demonstrated that PTRF knockdown/knockout increased apoptosis and decreased the proliferation of GBM cells when treated with TMZ. This finding is consistent with previous reports indicating that PTRF expression is necessary for multidrug resistance (MDR) in breast cancer cells 34 and increased GBM chemoresistance to imatinib 10. In addition, previous studies have reported that cancer cell-derived EVs could induce chemoresistance by reducing the intracellular accumulation of the drugs 8. EVs induced mesenchymal transition and therapeutic resistance in GBM through NF-κB/STAT3 signaling 35.Small EVs originate from the endosomal system through invagination of the plasma membrane and early endosome, which then mature into MVBs. The previous study had suggested PTRF overexpression increased the number of MVBs and exosome secretion. The increased MVBs may participate in the production of small EVs/exosome 6. These interesting findings made us investigate the role of EVs in TMZ resistance. Importantly, our results found that increased PTRF expression could induce intracellular TMZ efflux mediated by small EVs and large EVs, suggesting that PTRF can serve as an alternative drug target for which new therapies could be developed. Consistently, a previous study showed that elevated efflux of agents, which decreased the intracellular drug accumulation, became the major reason for chemo-resistance 36.

Currently, CQ is mainly used as an adjuvant that could sensitize certain types of tumor cells to antineoplastic drugs 37, 38. In this context, our *in vitro* and *in vivo* results strongly demonstrated that the sequential treatment of TMZ followed by CQ could inhibit glioma cell proliferation. Additionally, this sequential therapy elevated the [TMZ]i by reducing the secretion of EVs. Nonetheless, the [TMZ]i was not modulated in CQ+TMZ and CQ-TMZ groups compared to the administration of TMZ alone. This phenomenon might be explained considering the function of hydroxychloroquine (HCQ) in endocytosis inhibition. Previous study had reported CQ increased the pH in the lysosome and, as a result, the cell could not proceed with endocytosis, exosome release or phagolysosomal fusion 39, 40. Our findings are in agreement to previous reports showing that CQ effectively reversed MDR in non-small cell lung cancer cells by preventing and delaying the outward transport of antineoplastic drugs 17. Although we have demonstrated CQ treatment can decrease the expression of PTRF and caveolin1 in GBM, the detailed mechanism is still unclear and need further study. CQ exhibits rapid onset, long-acting half-life, low toxicity, high permeability of blood-brain barrier, and high tolerance in humans 41. These properties provide a safety guarantee for clinical applications of this type of sequential therapy in GBM patients.

In summary, the expression of PTRF is correlated with the survival time of GBM patients after TMZ treatment and proposed; this protein could function as a biomarker to predict the prognosis of GBM patients undergoing TMZ therapy. Furthermore, PTRF enhances TMZ-resistance by increasing the efflux of intracellular TMZ mediated by EVs. Moreover, the sequential therapy of TMZ followed by CQ could be key in overcoming TMZ-resistance by increasing intracellular concentrations of this agent, uncovering a novel idea for GBM treatment. Our findings could help in defining the usage of CQ and TMZ in future clinical trials.

## Supplementary Material

Supplementary figures and tables.

## Abbreviations

PTRF: polymerase I and transcript release factor
TMZ: temozolomide
GBM: glioblastoma
PTRF-ov: PTRF-eGFP
PDX: patient-derived xenograft
CQ: chloroquine
EVs: extracellular vesicles
DMEM: Dulbecco's modified Eagle medium
FBS: fetal bovine serum
PFA: paraformaldehyde
RIPA: radioimmunoprecipitation assay
PBST: phosphate buffered saline-Tween 20
TEM: Transmission Electron Microscopy
SEM: Scanning Electron Microscopy
HPLC: High-performance Liquid Chromatography
IHC: Immunohistochemical
DAB: diaminobenzidine
TUNEL: Terminal deoxynucleotidyl transferase dUTP Nick End Labeling
IF: Immunofluorescence
MGMT: O6-Methylguanine-DNA Methyltransferase
pMGMT: O6-Methylguanine-DNA Methyltransferase promoter
