# Supplementary material for: PTRF/Cavin-1 enhances chemo-resistance and promotes temozolomide efflux through extracellular vesicles in glioblastoma
Source: Theranostics. 2022 May 16;12(9):4330–47. doi: 10.7150/thno.71763 (PMC9169358; doi:10.7150/thno.71763)
Supplement: Supplementary file 1 — Supplementary figures and tables. [file thnov12p4330s1.pdf]

Supplementary

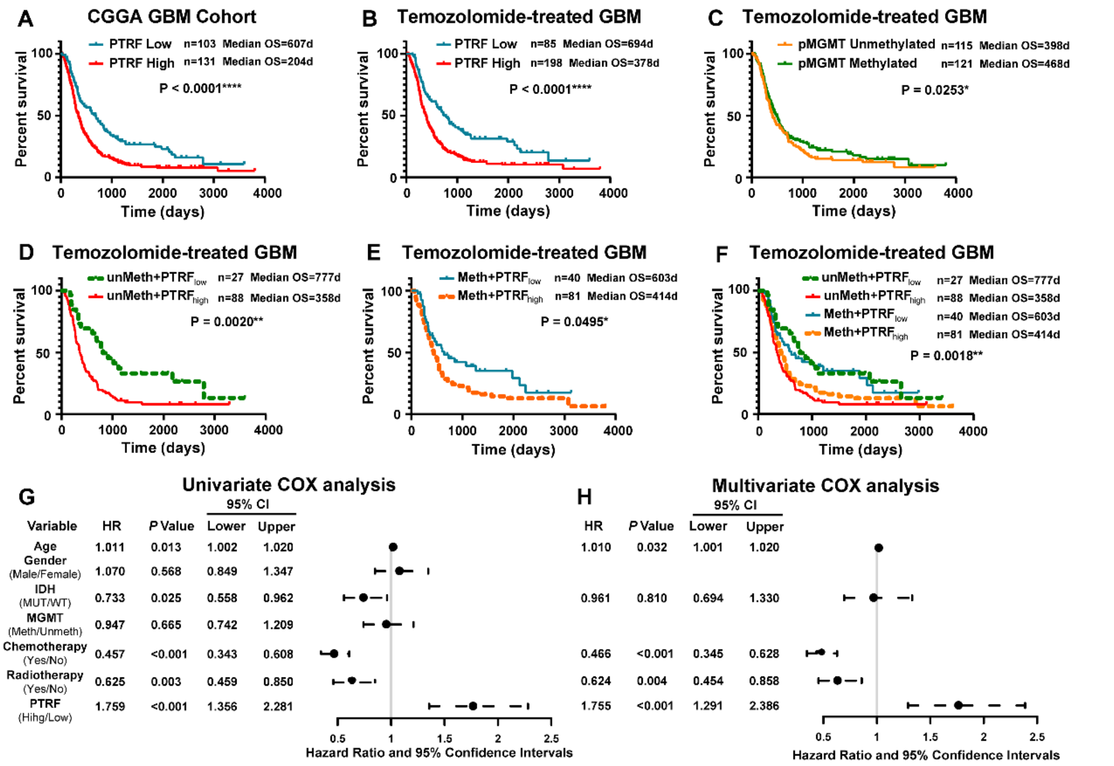

Figure S1. High PTRF expression confers a worse prognosis for TMZ treatment in GBM patients.

(A) Kaplan-Meier survival analysis of the expression level of PTRF in GBM patients based on microarray data. **(B)** Kaplan-Meier survival analysis of the expression levels of PTRF in TMZ-treated GBM patients based on microarray data. **(C)** Kaplan-Meier survival analysis of pMGMT unmethylated or methylated in TMZ-treated GBM patients based on microarray data. **(D-F)** Kaplan-Meier survival analysis of the effect of PTRF expression levels on pMGMT unmethylated or methylated TMZ-treated GBM patients. **(G-H)** Univariate and multivariate analyses of the PTRF expression and other clinical information in relation to overall survival in the CGGA GBM cohort.

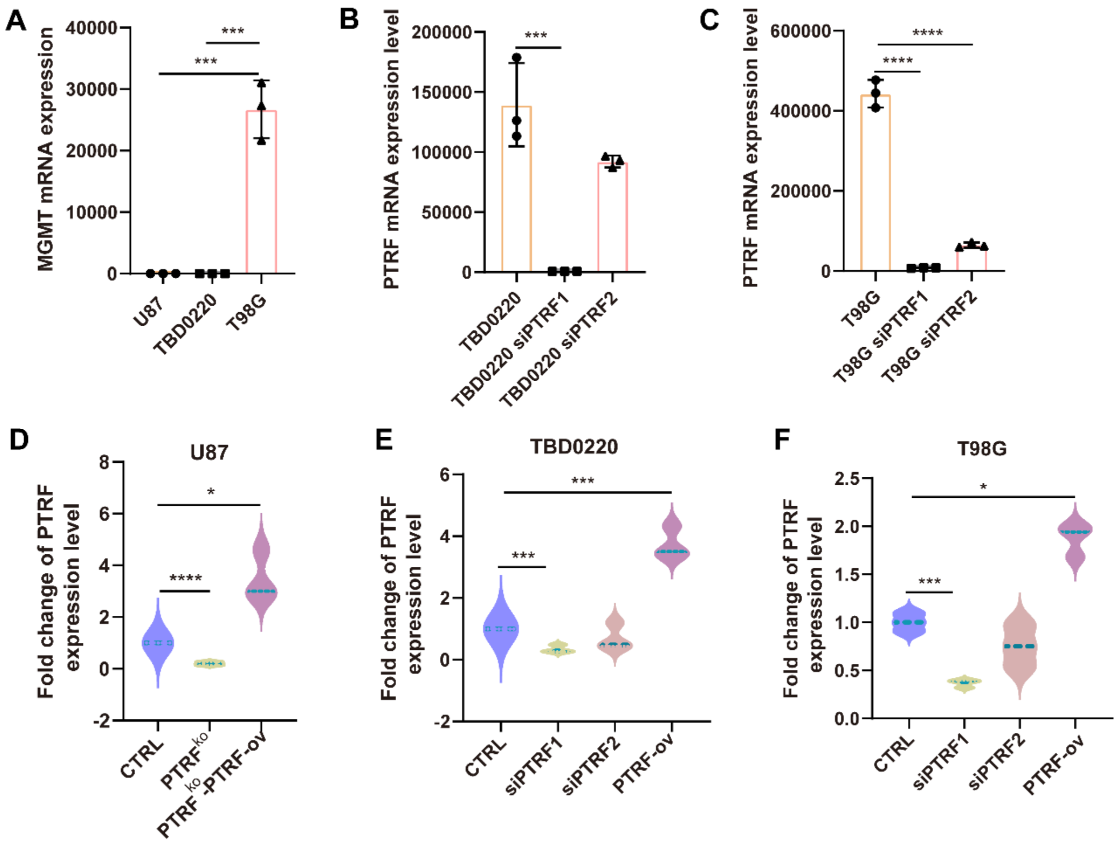

Figure S2. The expression of MGMT and PTRF.

(A) The mRNA expression of MGMT in U87, TBD0220, and T98G cells. Data are represented as the mean ± SEM (n = 3). ***p < 0.001. **(B-C)** The mRNA expression of PTRF in TBD0220 and T98G cells after different treatments. Data are represented as the mean ± SEM (n = 3). ***p < 0.001, ****p < 0.0001. **(D-F)** The protein expression level of PTRF in U87, TBD0220 and T98G cells after different treatments. Data are represented as the mean ± SEM (n = 3). *p < 0.05, ***p < 0.001, ****p < 0.0001.

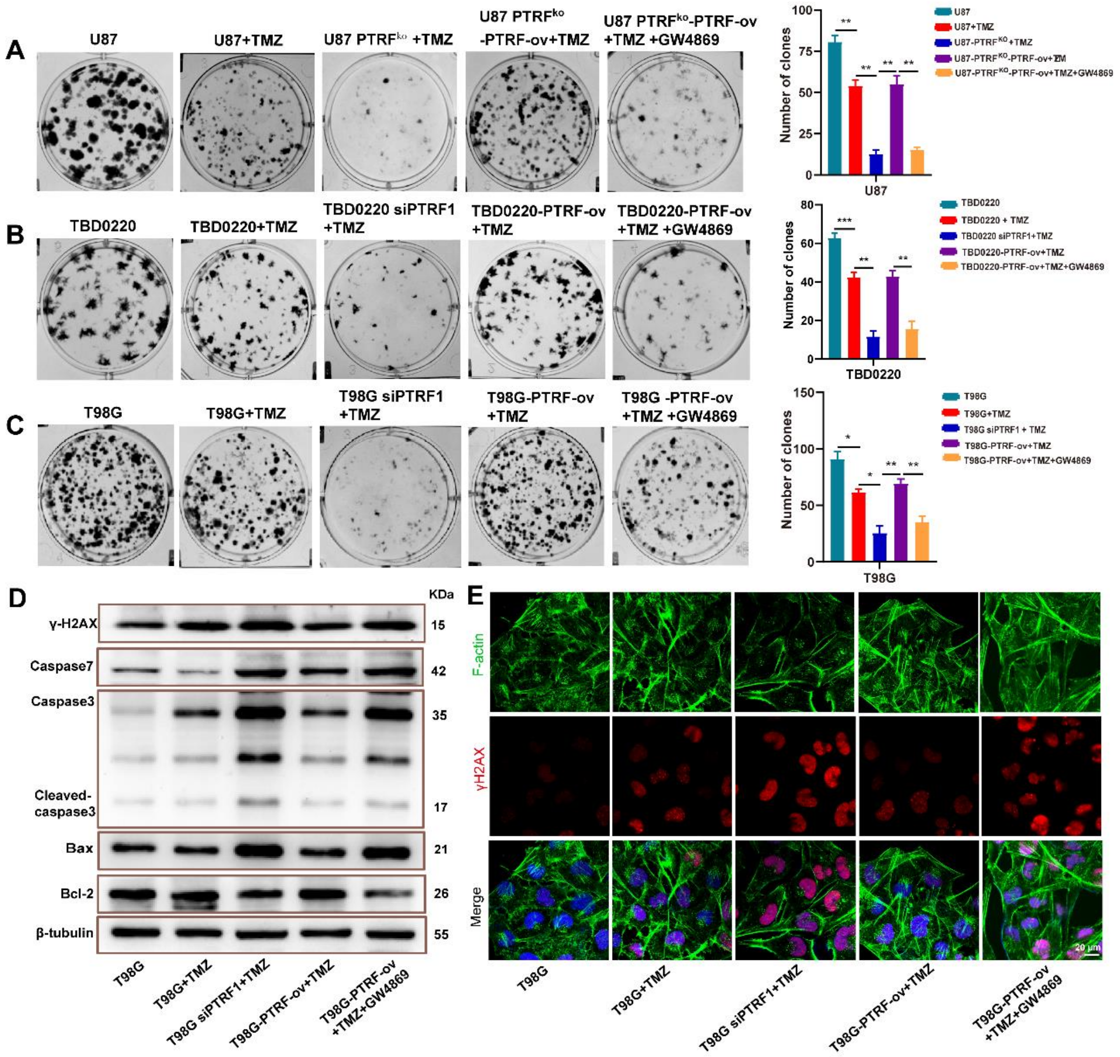

Figure S3. PTRF enhances TMZ resistance.

(A-C) Colony formation assay performed in U87, TBD0220 and T98G cells. Data are represented as the mean ± SEM (n = 3). *p < 0.05, **p < 0.01, ***p < 0.001. **(D)** Western blot analysis showing the protein expression of bax, bcl-2, caspase 3, caspase 7, and γH2AX after 800 μM TMZ treatment in T98G cells. **(E)** IF showing the expression of γH2AX after 800 μM TMZ treatment in T98G cells. Scale bar = 20 μm.

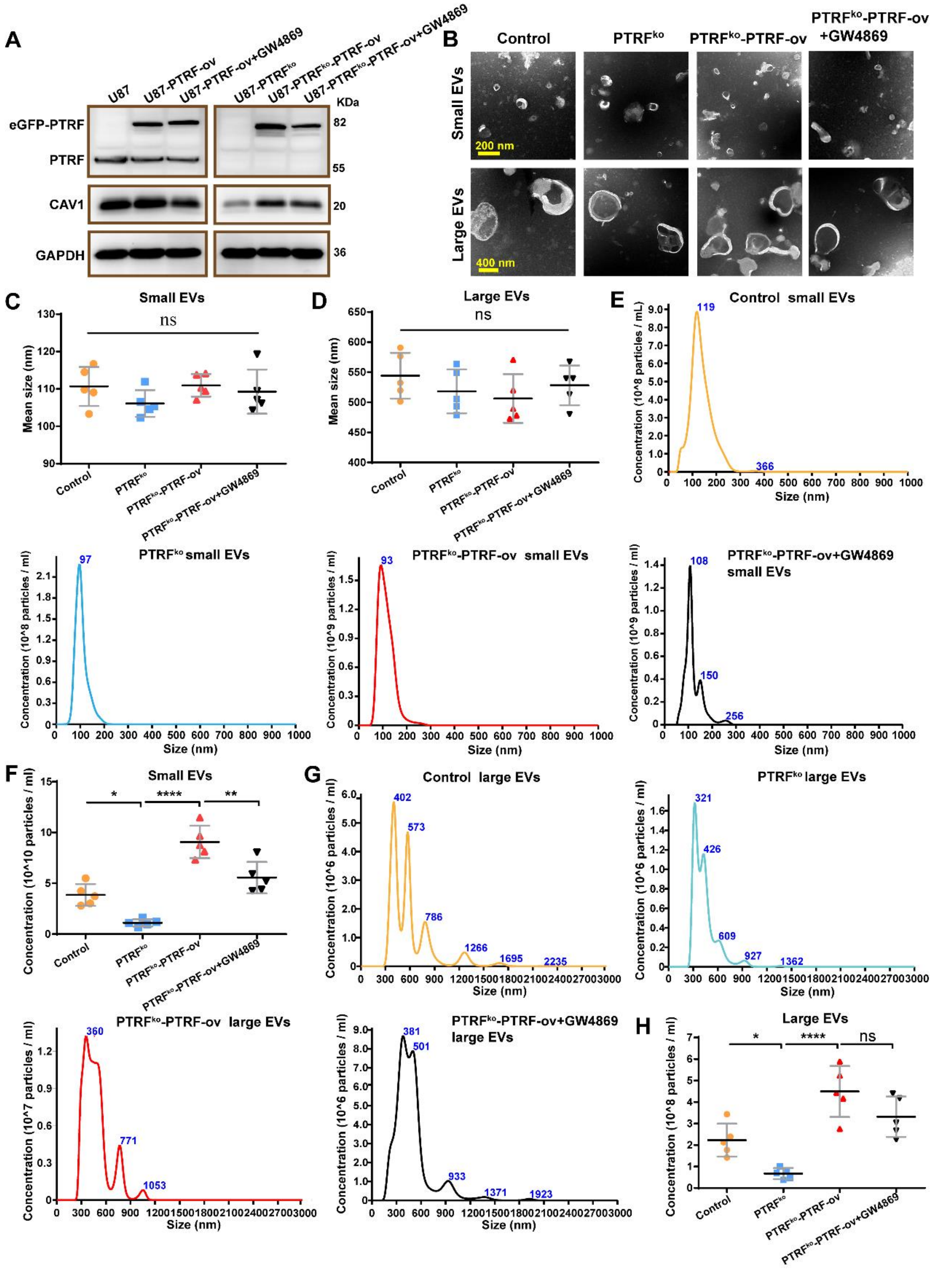

Figure S4: PTRF expression promotes the production of EVs.

(A) The expression of PTRF, CAV1, PTRF-eGFP under indicated treatments.

(B) Representative TEM images showing the morphologies of EVs after indicated

treatments. **(C-D)** NTA analysis showing the particle size distribution of EVs under indicated treatment. Data are represented as the mean ± SEM (n = 5). ns represents $p >$ 0.05. **(E-F)** NTA analysis showing the concentration of small EVs under indicated treatment. Data are represented as the mean ± SEM (n = 5). $*p < 0.05$, $**p < 0.01$, $****p$ < 0.0001. **(G-H)** NTA analysis showing the concentration of large EVs under indicated treatment. Data are represented as the mean ± SEM (n = 5). $*p < 0.05$, $****p < 0.0001$, ns represents p > 0.05.

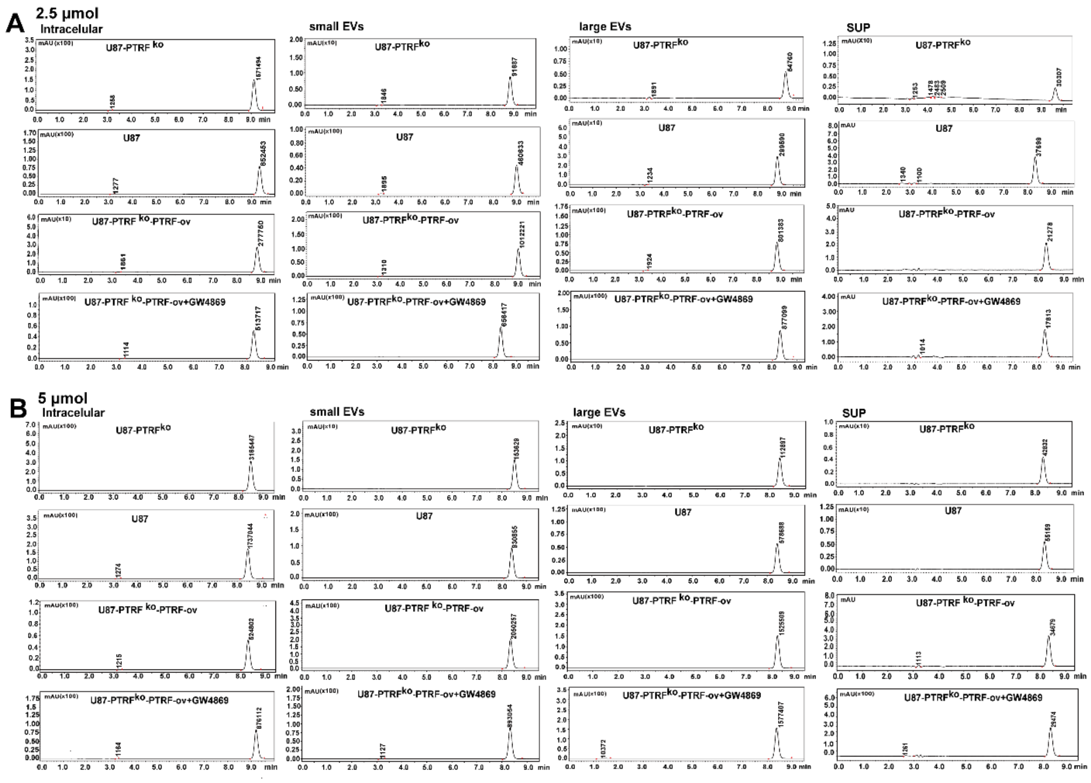

Figure S5: Knock out PTRF increases TMZ concentration in GBM cells.

(A) Chromatograms of U87 of 2.5 μmol TMZ administration.

(B) Chromatograms of U87 after 5 μmol TMZ administration.

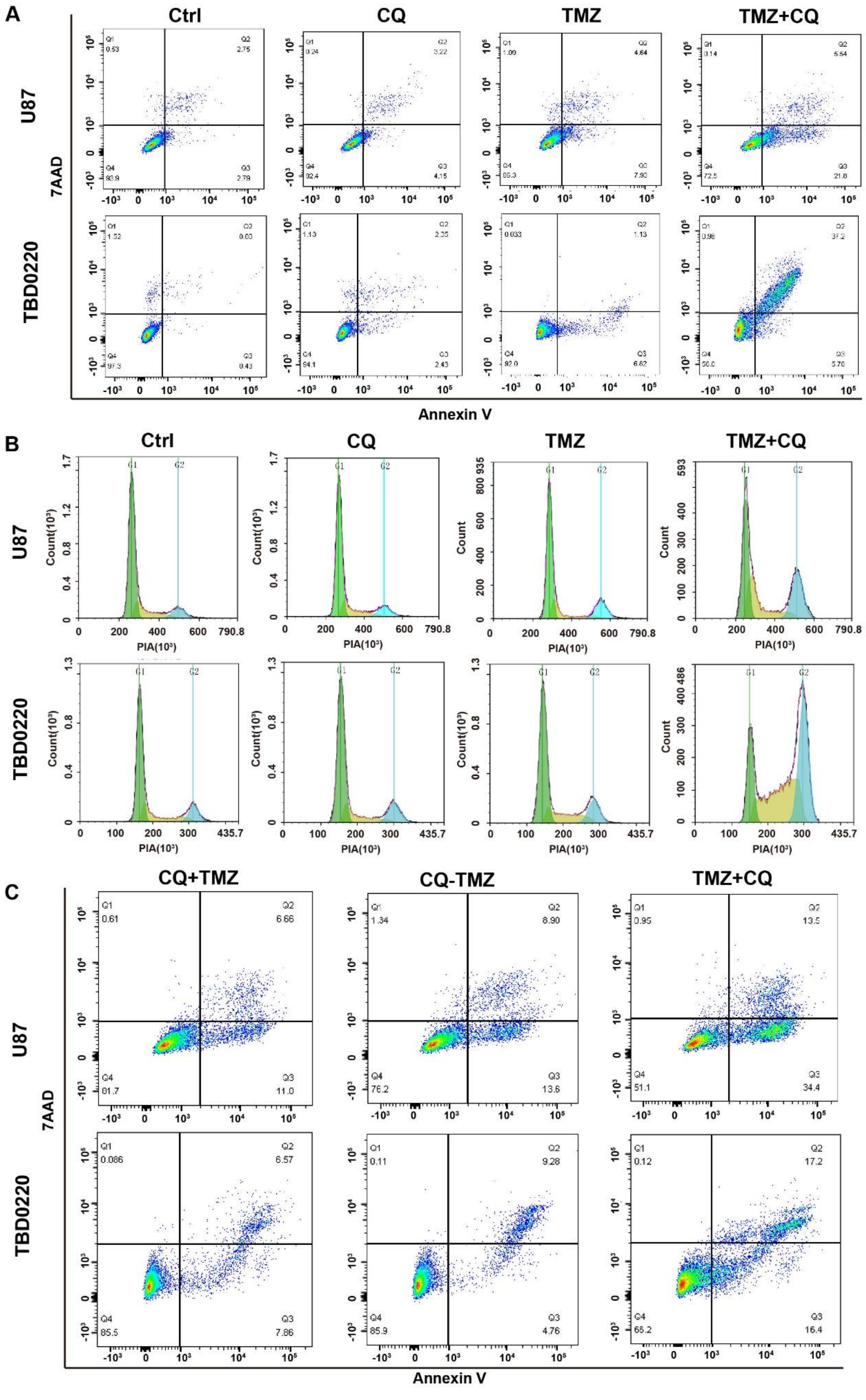

Figure S6: Sequential therapy of TMZ+CQ enhances TMZ efficacy in GBM.

(A) Flow cytometry analyses of Annexin V-FITC and 7-AAD staining after treating with 200 μM TMZ and/or 25 μM CQ. **(B)** Flow cytometry analysis of cell cycle after exposure to 200 μM TMZ and/or 25 μM CQ. **(C)** Flow cytometric analysis of Annexin V-FITC and 7-AAD staining in CQ+TMZ, CQ-TMZ, and TMZ+CQ treatment groups.

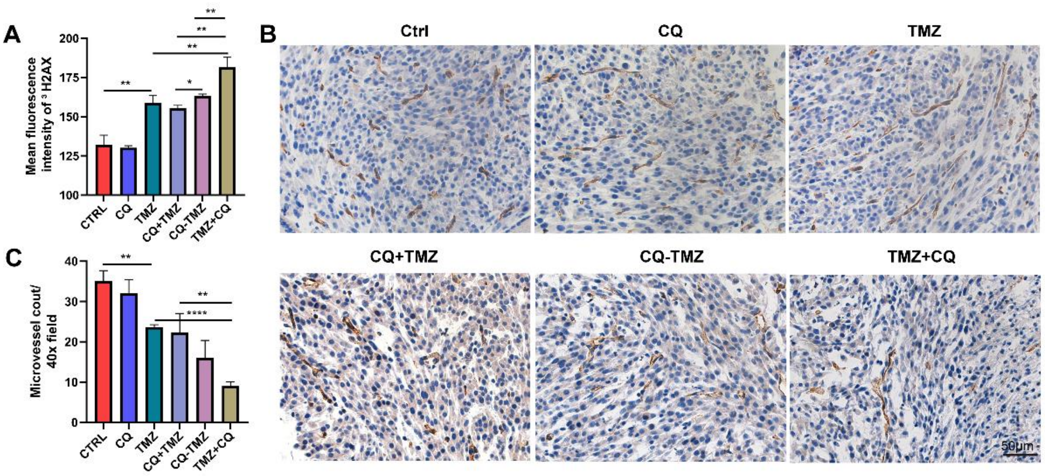

Figure S7: Sequential therapy of TMZ followed by CQ increased the efficacy of TMZ in orthotopic xenograft glioma mice

(A) IF of γH2AX expression in orthotopic xenograft glioma mice after different treatment. Data are represented as the mean ± SEM (n = 3). *$p < 0.05$, **$p < 0.01$. **(B-C)** IHC of CD31 expression in the brain tumor after different treatment. Data are represented as the mean ± SEM (n = 3). **$p < 0.01$, ****$p < 0.0001$. Scale bar = 50 μm.

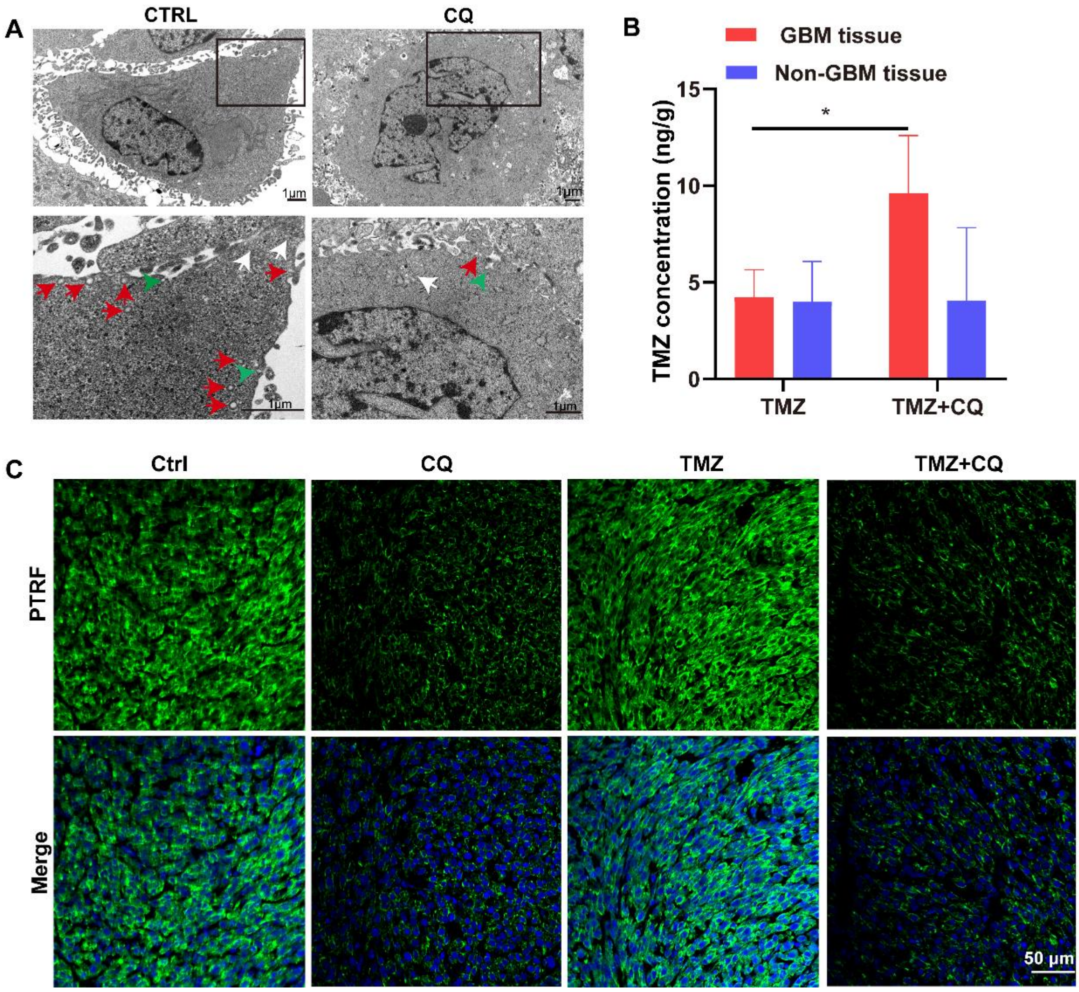

Figure S8: Sequential therapy of TMZ plus CQ increases intracellular TMZ concentration

(A) TEM analysis of vesicles, caveola, MVBs in control and CQ groups. Green arrowheads represent caveolae, red arrows represent endocytic vesicles and white arrows represent MVBs. Scale bar = 1 μm. **(B)** Sequential therapy of TMZ plus CQ increases the TMZ concentration in GBM tissue. Data are represented as the mean ± SEM (n = 3). $*p < 0.05$. **(C)** CQ treatment decreased the expression of PTRF in orthotopic xenograft glioma mice. Scale bar = 50 μm.

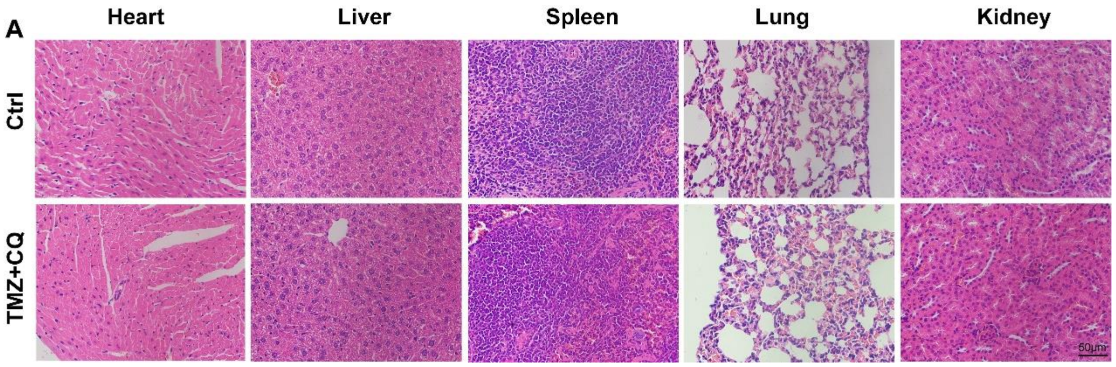

Figure S9: HE staining of the main organs of mice bearing orthotopic glioma

(A) Images of HE staining of the main organs of mice bearing orthotopic glioma treated with sequential therapy of TMZ plus CQ for 2 weeks. Scale bar: 50 μm

Table S1. The sequences of sgRNAs targeting PTRF.

Target	Catalog	Sequence
PTRF-sgRNA4	sgRNA#	TACCCGGAGCCGTGCGGGACCGG
PTRF-sgRNA6	sgRNA#	ATCAAGTCGGACCAGGTGAACGG

Table S2. The sequences of siRNAs targeting PTRF.

Target	Catalog	Sequence
siRNA (scramble)	siRNA#	5’-UUCUCCGAACGUGUCACGUTT-3’
PTRF1	siRNA#	5’-GCCGCAACUUUAAAGUCAUGAUCUA-3’
PTRF2	siRNA#	5’-AGGAGUCCCGCGCAGAGCGUAUCAA-3’

Table S3. The primers used for qRT-PCR.

**Target**		**Sequence**
GAPDH mRNA	Forward	GTCTCCTCTGACTTCAACAGCG
	Reverse	ACCACCCTGTTGCTGTAGCCAA
PTRF mRNA	Forward	GGCAGATCAAGAAGCTGGAGGT
	Reverse	CAGCGATTTGCTGATGCTCAGTT
MGMT mRNA	Forward	CCTGGCTGAATGCCTATTTCCAC
	Reverse	GCAGCTTCCATAACACCTGTCTG

TableS4.The IC50 values of TMZ treatment in the U87 after different treatments.

**Group**	**U87**	**U87-PTRF^{ko}**	**U87-PTRF^{ko}-PTRF-ov**	**U87-PTRF^{ko}-PTRF-ov+GW4869**
IC50 (μM)	345.7 μM	107.9 μM	842.4 μM	153.8 μM

TableS5. The IC50 values of TMZ treatment in the TBD0220 after different treatments.

**Group**	**TBD0220**	**TBD0220-siPTRF1**	**TBD0220-PTRF-ov**	**TBD0220-PTRF-ov+GW4869**
IC50 (μM)	213.4 μM	135.9 μM	596.5 μM	306 μM

TableS6. The IC50 values of TMZ treatment in the T98G after different treatments

**Group**	**T98G**	**T98G-siPTRF1**	**T98G-PTRF-ov**	**T98G-PTRF-ov+GW4869**
IC50 (μM)	1681 μM	1063 μM	1814 μM	1072 μM

Table S7. Blood routine analysis in blood samples of mice bearing glioma after 2 weeks continuous treatment

Group	WBC ($x10^9$/L)	RBC ($x10^{12}$/L)	HGB (g/L)	Plt ($x10^9$/L)
CTRL	5.0±1.3	10.4±0.78	155±15.62	1020±175.14
TMZ+CQ	10.13±2.15	9.1±0.21	134±5.29	1048±118.12

Table S8. Values of serum enzymes in blood samples of mice bearing orthotopic glioma after 2 weeks continuous treatment

Group	ALT (U/L)	AST (U/L)	ALP (U/L)	CR (μmol/L)	UA (μmol/L)	BUN (μmol/L)	ALB (g/l)
CTRL	92.4±6.49	319±139.18	261.1±31.293	45.4±7.37	270.93±44.95	13.96±3.18	27.8±0.964
TMZ+CQ	89.3±5.67	258.1±53.584	239.7±14.24	43.7±2.68	229.7±79.73	9.93±1.35	25.3±1.21
